# Integrative High Dimensional Multiple Testing with Heterogeneity under Data Sharing Constraints

**Published:** 2021-04

**Authors:** Molei Liu, Yin Xia, Kelly Cho, Tianxi Cai

**Affiliations:** Department of Biostatistics, Harvard T.H. Chan School of Public Health, USA; Department of Statistics, School of Management, Fudan University, China; Massachusetts Veterans Epidemiology Research and Information Center, US Department of Veteran Affairs, Brigham and Women’s Hospital, Harvard Medical School, USA; Department of Biostatistics, Harvard T.H. Chan School of Public Health, USA

**Keywords:** Debiasing, Distributed learning, False discovery rate, High dimensional inference, Integrative analysis, Multiple testing

## Abstract

Identifying informative predictors in a high dimensional regression model is a critical step for association analysis and predictive modeling. Signal detection in the high dimensional setting often fails due to the limited sample size. One approach to improving power is through meta-analyzing multiple studies which address the same scientific question. However, integrative analysis of high dimensional data from multiple studies is challenging in the presence of between-study heterogeneity. The challenge is even more pronounced with additional data sharing constraints under which only summary data can be shared across different sites. In this paper, we propose a novel data shielding integrative large–scale testing (DSILT) approach to signal detection allowing between-study heterogeneity and not requiring the sharing of individual level data. Assuming the underlying high dimensional regression models of the data differ across studies yet share similar support, the proposed method incorporates proper integrative estimation and debiasing procedures to construct test statistics for the overall effects of specific covariates. We also develop a multiple testing procedure to identify significant effects while controlling the false discovery rate (FDR) and false discovery proportion (FDP). Theoretical comparisons of the new testing procedure with the ideal individual–level meta–analysis (ILMA) approach and other distributed inference methods are investigated. Simulation studies demonstrate that the proposed testing procedure performs well in both controlling false discovery and attaining power. The new method is applied to a real example detecting interaction effects of the genetic variants for statins and obesity on the risk for type II diabetes.

## 1. Introduction

High throughput technologies such as genetic sequencing and natural language processing have led to an increasing number and types of predictors available to assist in predictive modeling. A critical step in developing accurate and robust prediction models is to differentiate true signals from noise. A wide range of high dimensional inference procedures have been developed in recent years to achieve variable selection, hypothesis testing and interval estimation (Van de Geer et al., 2014; Javanmard and Montanari, 2014; Zhang and Zhang, 2014; Chernozhukov et al., 2018, e.g.). However, regardless of the procedure, drawing precise high dimensional inference is often infeasible in practical settings where the available sample size is too small relative to the number of predictors. One approach to improve the precision and boost power is through meta-analyzing multiple studies that address the same underlying scientific problem. This approach has been widely adopted in practice in many scientific fields, including clinical trials, education, policy evaluation, ecology, and genomics (DerSimonian, 1996; Allen et al., 2002; Card et al., 2010; Stewart, 2010; Panagiotou et al., 2013, e.g.), as a tool for evidence-based decision making. Meta-analysis is particularly valuable in the high dimensional setting. For example, meta-analysis of high dimensional genomic data from multiple studies has uncovered new disease susceptibility loci for a broad range of diseases including Crohn’s disease, colorectal cancer, childhood obesity and type II diabetes (Houlston et al., 2008; Bradfield et al., 2012; Franke et al., 2010; Zeggini et al., 2008, e.g.).

Integrative analysis of high dimensional data, however, is highly challenging especially with biomedical studies for several reasons. First, between study heterogeneity arises frequently due to the difference in patient population and data acquisition. Second, due to privacy and legal constraints, individual level data often cannot be shared across study sites. Instead, only summary statistics can be passed between researchers. For example, patient level genetic data linked with clinical variables extracted from electronic health records (EHR) of several hospitals are not allowed to leave the firewall of each hospital. In addition to high dimensionality, attention to both heterogeneity and data sharing constraints are needed to perform meta-analysis of multiple EHR–linked genomic studies.

The aforementioned data sharing mechanism is referred to as DataSHIELD (Data aggregation through anonymous Summary–statistics from Harmonised Individual levEL Databases) in Wolfson et al. (2010), which has been widely accepted as a useful strategy to protect patient privacy (Jones et al., 2012; Doiron et al., 2013). Several statistical approaches to integrative analysis under the DataSHILED framework have been developed for low dimensional settings (Gaye et al., 2014; Zöller et al., 2018; Tong et al., 2020, e.g.). In the absence of cross-site heterogeneity, distributed high dimensional estimation and inference procedures have also been developed that can facilitate DataSHIELD constraints (Lee et al., 2017; Battey et al., 2018; Jordan et al., 2019, e.g.). Recently, Cai et al. (2019a) proposed an integrative high dimensional sparse regression approach that accounts for heterogeneity. However, their method is limited to parameter estimation and variable selection. To the best of our knowledge, no hypothesis testing procedures currently exist to enable identification of significant predictors with false discovery error control under the setting of interest. In this paper, we propose a data shielding integrative large–scale testing (DSILT) procedure to fill this gap.

### 1.1 Problem statement

Suppose there are M independent studies and the mth study contains observations on an outcome $Y^{\left(\text{m}\right)}$ and a p-dimensional covariate vector $X^{\left(\text{m}\right)}$, where $Y^{\left(\text{m}\right)}$ can be binary or continuous, and without loss of generality we assume that $X^{\left(\text{m}\right)}$ contains 1 as its first element. Specifically, data from the mth study consist of $n_{m}$ independent and identically distributed random vectors, ${𝓓}^{\left(\text{m}\right)}=\left\{D_{i}^{\left(\text{m}\right)}={\left(Y_{i}^{\left(\text{m}\right)},X_{i}^{(\text{m})⊤}\right)}^{⊤},i=1,\ldots ,n_{m}\right\}$. Let $N=\sum_{m=1}^{M}n_{m}$ and n=N/M. We assume a conditional mean model $\text{E}\left(Y^{\left(\text{m}\right)}|X^{\left(\text{m}\right)}\right)=g\left(\beta_{0}^{(\text{m})⊤}X^{\left(\text{m}\right)}\right)$ and that the true model parameter $\beta_{0}^{\left(\text{m}\right)}$ is the minimizer of the population loss function:

$$\beta_{0}^{\left(\text{m}\right)}=\underset{\beta^{\left(\text{m}\right)}\in {\mathbb{R}}^{p}}{\text{argmin}}{𝓛}_{m}\left(\beta^{\left(\text{m}\right)}\right),\;\text{where}\;{𝓛}_{m}\left(\beta^{\left(\text{m}\right)}\right)=\text{E}\left\{f\left(X_{i}^{(\text{m})\text{T}}\beta^{\left(\text{m}\right)},Y_{i}^{\left(\text{m}\right)}\right)\right\},\;f(x,y)=\phi (x)-yx,$$

where $\dot{\phi }(x)\equiv d\phi (x)/dx=g(x)$. When $\phi (x)=\log\left(1+e^{x}\right)$, this corresponds to a logistic model if Y is binary and a quasi-binomial model if Y∈[0,1] is a continuous probability score sometimes generated from an EHR probabilistic phenotyping algorithm. One may take $\phi (x)=e^{x}$ for some non-negative Y such as the count (or log-count) of a diagnostic code in EHR studies ^1^. As detailed in Assumptions 2-3 of Section 3.1, our procedure allows for a broad range of models provided that g(⋅) is smooth and the residuals $Y_{i}^{\left(\text{m}\right)}-g\left(\beta_{0}^{(\text{m})⊤}X_{i}^{\left(\text{m}\right)}\right)$ are sub-Gaussian, although not all generalized linear models satisfy these assumptions.

Under the DataSHIELD constraints, the individual–level data ${𝓓}^{\left(\text{m}\right)}$ is stored at the $m^{\text{th}}$ data computer (DC) and only summary statistics are allowed to transfer from the distributed DCs to the analysis computer (AC) at the central node. Our goal is to develop procedures under the DataSHIELD constraints for testing

(1) $$H_{0,j}:\beta_{0,j}\equiv {\left(\beta_{0,j}^{\left(1\right)},\ldots ,\beta_{0,j}^{\left(\text{M}\right)}\right)}^{⊤}=0\;\text{v.s.}\;H_{a,j}:\beta_{0,j}\neq 0$$

simultaneously for j∈𝓗 to identify ${𝓗}_{1}=\left\{j\in 𝓗:\beta_{0,j}\neq 0\right\}$, while controlling the false discovery rate (FDR) and false discovery proportion (FDP), where 𝓗⊆{2,…,p} is a user-specified subset with |𝓗|=q≍p and |𝓐| denotes the size of any set 𝓐. Here $\beta_{0,j}=0$ indicates that $X_{j}$ is independent of Y given all remaining covariates. To ensure effective integrative analysis, we assume that $\beta_{0}^{\left(1\right)},\ldots ,\beta_{0}^{\left(\text{M}\right)}$ are sparse and share similar support. Specifically, we assume that $\left|{𝓢}_{0}\right|\ll p$ and, $s^{\left(\text{m}\right)}≍s$ for m=1,2,…,M, where ${𝓢}_{0}=\left\{j=2,\ldots ,p:\beta_{0,j}^{\left(\text{m}\right)}\neq 0\right\}=\cup_{m=1}^{M}{𝓢}^{\left(\text{m}\right)}$, ${𝓢}^{\left(\text{m}\right)}=\left\{j=2,\ldots ,p:\beta_{0,j}^{\left(\text{m}\right)}\neq 0\right\}$, $s^{\left(\text{m}\right)}=\left|{𝓢}^{\left(\text{m}\right)}\right|$, and $s=\left|{𝓢}_{0}\right|$.

### 1.2 Our contribution and the related work

We propose in this paper a novel DSILT procedure with FDR and FDP control for the simultaneous inference problem (1). The proposed testing procedure consists of three major steps: (I) derive an integrative estimator on the AC using locally obtained summary statistics from the DCs and send the estimator back to the DCs; (II) construct a group effect test statistic for each covariate through an integrative debiasing method; and (III) develop an error rate controlled multiple testing procedure based on the group effect statistics.

The integrative estimation approach in the first step is closely related to the group inference methods in the literature. Denote by $\beta_{j}={\left(\beta_{j}^{\left(1\right)},\ldots ,\beta_{j}^{\left(\text{M}\right)}\right)}^{⊤}$, $\beta^{\left(•\right)}={\left(\beta^{(1)⊤},\ldots ,\beta^{(\text{M})⊤}\right)}^{\text{T}}$,

$${\hat{𝓛}}^{\left(\text{m}\right)}\left(\beta^{\left(\text{m}\right)}\right)=n_{m}^{-1}\sum_{i=1}^{n_{m}}f\left(\beta^{(\text{m})\text{T}}X_{i}^{\left(\text{m}\right)},Y_{i}^{\left(\text{m}\right)}\right)\;\text{and}\;{\hat{𝓛}}^{\left(•\right)}\left(\beta^{\left(•\right)}\right)=N^{-1}\sum_{m=1}^{M}n_{m}{\hat{𝓛}}_{m}\left(\beta^{\left(\text{m}\right)}\right).$$

Literature in group LASSO and multi-task learning (Huang and Zhang, 2010; Lounici et al., 2011, e.g.) established that, under the setting $s^{\left(\text{m}\right)}≍s$ as introduced in Section 1.1, the group LASSO estimator with tuning parameter $\lambda :{\text{argmin}}_{\beta (•)}\;{\hat{𝓛}}^{\left(•\right)}\left(\beta^{\left(•\right)}\right)+\lambda \sum_{j=2}^{p}{\left\|\beta_{j}\right\|}_{2}$, benefits from the group structure and attains the optimal rate of convergence. In this paper, we adopt the same structured group LASSO penalty for integrative estimation, but under data sharing constraints. Recently, Mitra et al. (2016) proposed a group structured debiasing approach under the integrative analysis setting, where they restricted their analysis to linear models and required that the precision matrices of the covariates be group-sparse across the distributed datasets. In contrast, our method accommodates non-linear models and imposes no sparsity or homogeneity structures on the covariate distributions from different local sites (see Assumption 1 in Section 3.1).

The second step of our method, i.e., the construction of the test statistics for each of the hypotheses, relies on the group debiasing of the above integrative estimation. For debiasing of M–estimation, nodewise LASSO regression was employed in the earlier work (Van de Geer et al., 2014; Janková and Van De Geer, 2016, e.g), while the Dantzig selector type approach was proposed more recently (Belloni et al., 2018; Caner and Kock, 2018, e.g). We develop in this article a cross–fitted group Dantzig selector type debiasing method, which requires weaker inverse Hessian assumptions (see Assumption 1 in Section 3.1) than the aforementioned approaches. In addition, the proposed debiasing step achieves proper bias rate under the same model sparsity assumptions as the ideal individual–level meta–analysis (ILMA) method. Compared with the One–shot distributed inference approaches (Tang et al., 2016; Lee et al., 2017; Battey et al., 2018), the proposed method additionally considers model heterogeneity and group inference; it further reduces the bias rate by sending the integrative estimator to the DCs to derive updated summary statistics, which in turn benefits the subsequent multiple testing procedure. See Section 3.4 for detailed comparisons.

As the last step, simultaneous inference with theoretical error rates control is performed based on the group effect statistics. The test statistics are shown to be asymptotically chi-square distributed under the null, and the proposed multiple testing procedure asymptotically controls both the FDR and FDP at the pre-specified level. Multiple testing for high dimensional regression models has recently been studied in the literature (Liu and Luo, 2014; Xia et al., 2018b,a; Javanmard et al., 2019, e.g). Our testing step for FDR control as a whole differs considerably from these existing procedures in the following aspects. First, the proposed test statistics, the key input to the FDR control procedure, are brand new and the resulting estimation of false discovery proportion differs fundamentally from those of the literature. Second, we consider a more general M–estimation setting which can accommodate different types of outcomes. Third, we allow the heterogeneity in both the covariates and the coefficients. Fourth, the existing testing approaches developed for individual-level data are not suitable for the DataSHIELD framework. Last, because there are complicated dependence structures among the integrative chi-squared statistics under the DataSHIELD constraints, the theoretical derivations are technically much more involved. Hence, our proposal makes a useful addition to the general toolbox of simultaneous regression inference.

We demonstrate here via numerical experiments that the proposed DSILT procedure attains good power while maintaining error rate control. In addition, we demonstrate how our new approach outperforms existing distributed inference methods and enjoys similar performance as the ideal ILMA approach.

### 1.3 Outline of the paper

The rest of this paper is organized as follows. We detail the DSILT approach in Section 2. In Section 3, we present asymptotic analysis on the false discovery control of our method and compare it with the ILMA and One–shot approach. In Section 4, we summarize finite sample performance of our approach along with other methods from simulation studies. In Section 5, we apply our proposed method to a real example. Proofs of the theoretical results and additional technical lemmas and simulation results are collected in the Supplementary Material.

## 2. Data shielding integrative large–scale testing procedure

In this section, we study the detailed procedure of the proposed method. We start with some notation that will be used throughout the paper.

### 2.1 Notation

For any integer d, any vector $x={\left(x_{1},x_{2},\ldots ,x_{d}\right)}^{⊤}\in {\mathbb{R}}^{d}$, and any set $𝓢=\left\{j_{1},\ldots ,j_{k}\right\}\subseteq [d]\equiv \{1,\ldots ,d\}$, denote by $x_{𝓢}={\left[x_{j_{1}},\ldots ,x_{j_{k}}\right]}^{\prime }$, $x_{-j}$ the vector with its $j^{\text{th}}$ entry removed from x, $∥x{∥}_{q}$ the $\ell_{q}$ norm of x and $∥x{∥}_{\infty }=\max_{j\in [d]}\left|x_{j}\right|$. For any d-dimensional vectors $\{a^{\left(\text{m}\right)}={\left(a_{1}^{\left(\text{m}\right)},\ldots ,a_{d}^{\left(\text{m}\right)}\right)}^{⊤},m\in [M]\}$ and 𝓢⊆[d], let $a^{\left(•\right)}={\left(a^{(1)⊤},\ldots ,a^{(\text{M})⊤}\right)}^{⊤}$, $a_{𝓢}^{\left(•\right)}={\left(a_{𝓢}^{(1)⊤},\ldots ,a_{𝓢}^{(\text{M})⊤}\right)}^{⊤}$, $a_{j}={\left(a_{j}^{\left(1\right)},\ldots ,a_{j}^{\left(\text{M}\right)}\right)}^{⊤}$, and $a_{-j}^{\left(•\right)}={\left(a_{-j}^{(1)⊤},\ldots ,a_{-j}^{(\text{M})⊤}\right)}^{⊤}$. Let $e_{j}$ be the unit vector with $j^{\text{th}}$ element being 1 and remaining elements being 0 and $e_{j}^{\left(•\right)}={\left(e_{j}^{⊤},\ldots ,e_{j}^{⊤}\right)}^{⊤}$. Denote by ${\left\|a^{\left(•\right)}\right\|}_{2,1}=\sum_{j=1}^{d}{\left\|a_{j}\right\|}_{2}$ and ${\left\|a^{\left(•\right)}\right\|}_{2,\infty }=\max_{j\in [d]}{\left\|a_{j}\right\|}_{2}$ the $\ell_{2}/\ell_{1}$ and $\ell_{2}/\ell_{\infty }$ norm of $a^{\left(•\right)}$ respectively. For any K–fold partition of $\left[n_{m}\right]$, denoted by $\left\{{𝓘}_{k}^{\left(\text{m}\right)},k\in [K]\right\}$, let ${𝓘}_{-k}^{\left(\text{m}\right)}=\left[n_{m}\right]\setminus {𝓘}_{k}^{\left(\text{m}\right)}$, ${𝓘}_{k}^{\left(•\right)}=\left\{{𝓘}_{k}^{\left(\text{m}\right)}:m\in [M]\right\}$, ${𝓘}_{-k}^{\left(•\right)}=\left\{{𝓘}_{-k}^{\left(\text{m}\right)}:m\in [M]\right\}$. For any index set ${𝓘}^{\left(•\right)}=\{{𝓘}^{\left(\text{m}\right)}\subseteq \left[n_{m}\right],m\in [M]\}$, ${𝓓}_{{𝓘}^{\left(\text{m}\right)}}^{\left(\text{m}\right)}=\left\{D_{i}^{\left(\text{m}\right)}:i\in {𝓘}^{\left(\text{m}\right)}\right\}$ and ${𝓓}_{{𝓘}^{\left(•\right)}}^{\left(•\right)}=\left\{{𝓓}_{{𝓘}^{\left(\text{m}\right)}}^{\left(\text{m}\right)}:m\in [M]\right\}$. Let $\ddot{\phi }(\theta )=d^{2}\phi (\theta )/d\theta^{2}\geq 0$. Denote by $\beta_{0,j}$ and $\beta_{0}^{\left(•\right)}$ the true values of $\beta_{j}$ and $\beta^{\left(•\right)}$ respectively. For any ${𝓘}^{\left(•\right)}$ and $\beta^{\left(•\right)}$, define the sample measure operators ${\hat{𝓟}}_{{𝓘}^{\left(\text{m}\right)}}\eta_{\beta^{\left(\text{m}\right)}}={\left|{𝓘}^{\left(\text{m}\right)}\right|}^{-1}\sum_{i\in {𝓘}^{\left(\text{m}\right)}}\eta_{\beta^{\left(\text{m}\right)}}\left(D_{i}^{\left(\text{m}\right)}\right)$ and ${\hat{𝓟}}_{{𝓘}^{\left(•\right)}}\eta_{\beta^{\left(•\right)}}={\left|{𝓘}^{\left(•\right)}\right|}^{-1}\sum_{m=1}^{M}\sum_{i\in {𝓘}^{\left(\text{m}\right)}}\eta_{\beta^{\left(\text{m}\right)}}\left(D_{i}^{\left(\text{m}\right)}\right)$, and the population measure operator ${𝓟}^{\left(\text{m}\right)}\eta_{\beta^{\left(\text{m}\right)}}=\text{E}\eta_{\beta^{\left(\text{m}\right)}}\left(D_{i}^{\left(\text{m}\right)}\right)$, for all integrable functions $\eta_{\beta (•)}=\left\{\eta_{\beta^{\left(\text{m}\right)}},m\in [M]\right\}$ parameterized by $\beta^{\left(•\right)}$ or $\beta^{\left(\text{m}\right)}$.

For any given $\beta^{\left(\text{m}\right)}$, we define $\theta_{i}^{\left(\text{m}\right)}=X_{i}^{(\text{m})⊤}\beta^{\left(\text{m}\right)}$, $\theta_{0,i}^{\left(\text{m}\right)}=X_{i}^{(\text{m})\text{T}}\beta_{0}^{\left(\text{m}\right)}$, and the residual $\epsilon_{i}^{\left(\text{m}\right)}:=Y_{i}^{\left(\text{m}\right)}-\dot{\phi }\left(\theta_{0,i}^{\left(\text{m}\right)}\right)$. Similar to Cai et al. (2019b) and Ma et al. (2020), given coefficient $\beta^{\left(\text{m}\right)}$, we can express $Y_{i}^{\left(\text{m}\right)}∼X_{i}^{\left(\text{m}\right)}$ in an approximately linear form:

$$Y_{i}^{\left(\text{m}\right)}-\dot{\phi }\left(\theta_{i}^{\left(\text{m}\right)}\right)+\ddot{\phi }\left(\theta_{i}^{\left(\text{m}\right)}\right)\theta_{i}^{\left(\text{m}\right)}=\ddot{\phi }\left(\theta_{i}^{\left(\text{m}\right)}\right)X_{i}^{(\text{m})\text{T}}\beta_{0}^{\left(\text{m}\right)}+\epsilon_{i}^{\left(\text{m}\right)}+R_{i}^{\left(\text{m}\right)}\left(\theta_{i}^{\left(\text{m}\right)}\right),$$

where $R_{i}^{\left(\text{m}\right)}\left(\theta_{i}^{\left(\text{m}\right)}\right)$ is the reminder term and $R_{i}^{\left(\text{m}\right)}\left(\theta_{0,i}^{\left(\text{m}\right)}\right)=0$. For a given observation set D and coefficient β, we let $\theta =X^{⊤}\beta$, $Y_{\beta }={\ddot{\phi }}^{-\frac{1}{2}}(\theta )\{Y-\dot{\phi }(\theta )+\ddot{\phi }(\theta )\theta \}$, $X_{\beta }={\ddot{\phi }}^{\frac{1}{2}}(\theta )X$. Note that for the logistic model, we have $\text{Var}\left(Y_{\beta }|X_{\beta }\right)=1$, and $X_{\beta }$ and $Y_{\beta }$ can be viewed as the covariates and responses adjusted for the heteroscedasticity of the residuals.

### 2.2 Outline of the proposed testing procedure

We first outline in this section the DSILT procedure in Algorithm 1 and then study the details of each key step later in Sections 2.3 to 2.5. The procedure involves partitioning of ${𝓓}^{\left(\text{m}\right)}$ into K folds $\left\{{𝓘}_{k}^{\left(\text{m}\right)}:k\in [K]\right\}$ for m∈[M], where without loss of generality we let K≥2 be an even number. With a slight abuse of notation, we write ${𝓓}_{\left[k\right]}^{\left(\text{m}\right)}={𝓓}_{{𝓘}_{k}^{\left(\text{m}\right)}}^{\left(\text{m}\right)}$, ${𝓓}_{\left[k\right]}^{\left(•\right)}={𝓓}_{{𝓘}_{k}^{\left(•\right)}}^{\left(•\right)}$, ${𝓓}_{\left[-k\right]}^{\left(\text{m}\right)}={𝓓}_{{𝓘}_{-k}^{\left(\text{m}\right)}}^{\left(\text{m}\right)}$, and ${𝓓}_{\left[-k\right]}^{\left(•\right)}={𝓓}_{{𝓘}_{-k}^{\left(•\right)}}^{\left(•\right)}$.

**Algorithm 1:** DSILT Algorithm.

Input: ${𝓓}^{\left(\text{m}\right)}$ at the $m^{\text{th}}$ DC for m∈[M].	
1.	For each k∈[K], fit **integrative sparse regression under DataSHIELD** with ${𝓓}_{\left[-k\right]}^{\left(•\right)}$:
	(a) At the $m^{\text{th}}$ DC, construct cross-fitted summary statistics based on local LASSO estimator, and send them to the AC;
	(b) Obtain the integrative estimator ${\tilde{\beta }}_{\left[-k\right]}^{\left(•\right)}$ at AC and send them back to each DC.
2.	**Obtain debiased group test statistics**:
	(a) For each k, at the $m^{\text{th}}$ DC, obtain the updated summary statistics based on ${\tilde{\beta }}_{\left[-k\right]}^{\left(•\right)}$ and ${𝓓}_{\left[k\right]}^{\left(\text{m}\right)}$, and send them to the AC;
	(b) At the AC, construct cross-fitted debiased group estimators $\left\{{\overset{˘}{\zeta }}_{j},j\in 𝓗\right\}$.
3.	Construct a multiple testing procedure based on the test statistics from Step 2.

### 2.3 Step 1: Integrative sparse regression

As a first step, we fit integrative sparse regression under DataSHIELD with ${𝓓}_{\left[-k\right]}^{\left(•\right)}$ following similar strategies as given in Cai et al. (2019a). To carry out Step 1(a) of Algorithm 1, we split the index set ${𝓘}_{-k}^{\left(\text{m}\right)}$ into $K^{\prime }$ folds ${𝓘}_{-k,1}^{\left(\text{m}\right)},\ldots ,{𝓘}_{-k,K^{\prime }}^{\left(\text{m}\right)}$. For k∈[K] and $k^{\prime }\in \left[K^{\prime }\right]$, we construct local LASSO estimator with tuning parameter $\lambda^{\left(\text{m}\right)}:{\hat{\beta }}_{\left[-k,-k^{\prime }\right]}^{\left(\text{m}\right)}={\text{argmin}}_{\beta^{\left(\text{m}\right)}\in {\mathbb{R}}^{p}}{\hat{𝓟}}_{{𝓘}_{-k}^{\left(\text{m}\right)}\setminus {𝓘}_{-k,k^{\prime }}^{\left(\text{m}\right)}}f\left(X^{⊤}\beta^{\left(\text{m}\right)},Y\right)+\lambda^{\left(\text{m}\right)}{\left\|\beta_{-1}^{\left(\text{m}\right)}\right\|}_{1}$. With ${𝓓}_{\left[-k\right]}^{\left(\text{m}\right)}$, we then derive summary data ${𝓢}_{\left[-k\right]}^{\left(\text{m}\right)}=\left\{\left|{𝓘}_{-k}^{\left(\text{m}\right)}\right|,{\hat{\xi }}_{\left[-k\right]}^{\left(\text{m}\right)},{\hat{ℍ}}_{\left[-k\right]}^{\left(\text{m}\right)}\right\}$, where

(2) $${\hat{\xi }}_{\left[-k\right]}^{\left(\text{m}\right)}=K^{\prime -1}\sum_{k^{\prime }=1}^{K^{\prime }}{\hat{𝓟}}_{{𝓘}_{-k,k^{\prime }}^{\left(\text{m}\right)}}X_{{\hat{\beta }}_{\left[-k,-k^{\prime }\right]}^{\left(\text{m}\right)}}Y_{{\hat{\beta }}_{\left[-k,-k^{\prime }\right]}^{\left(\text{m}\right)}},\;{\hat{ℍ}}_{\left[-k\right]}^{\left(\text{m}\right)}=K^{\prime -1}\sum_{k^{\prime }=1}^{K^{\prime }}{\hat{𝓟}}_{{𝓘}_{-k,k^{\prime }}^{\left(\text{m}\right)}}X_{{\hat{\beta }}_{\left[-k,-k^{\prime }\right]}^{\left(\text{m}\right)}}X_{{\hat{\beta }}_{\left[-k,-k^{\prime }\right]}^{\left(\text{m}\right)}}^{⊤}.$$

In Step 1(b) of Algorithm 1, for k∈[K], we aggregate the M sets of summary data $\left\{{𝓢}_{\left[-k\right]}^{\left(\text{m}\right)},m\in [M]\right\}$ at the central AC and solve a regularized quasi–likelihood problem to obtain the integrative estimator with tuning parameter λ:

(3) $${\tilde{\beta }}_{\left[-k\right]}^{\left(•\right)}=\underset{\beta^{\left(•\right)}}{\text{argmin}}{\left|{𝓘}_{-k}^{\left(•\right)}\right|}^{-1}\sum_{m=1}^{M}\left|{𝓘}_{-k}^{\left(\text{m}\right)}\right|\left(\beta^{(\text{m})\text{T}}{\hat{ℍ}}_{\left[-k\right]}^{\left(\text{m}\right)}\beta^{\left(\text{m}\right)}-2\beta^{(\text{m})\text{T}}{\hat{\xi }}_{\left[-k\right]}^{\left(\text{m}\right)}\right)+\lambda {\left\|\beta_{-1}^{\left(•\right)}\right\|}_{2,1}.$$

These K sets of estimators, $\left\{{\tilde{\beta }}_{\left[-k\right]}^{\left(•\right)},k\in [K]\right\}$, are then sent back to the DCs. The summary statistics introduced in (2) can be viewed as the covariance terms of ${𝓓}_{\left[-k\right]}^{\left(\text{m}\right)}$ with the local LASSO estimator plugged-in to adjust for the heteroscedasticity of the residuals. Cross–fitting is used to remove the dependence of the observed data and the fitted outcomes - a strategy frequently employed in high dimensional inference literatures (Chernozhukov et al., 2016, 2018). As in Cai et al. (2019a), the integrative procedure can also be viewed in such a way that $\beta^{(\text{m})⊤}{\hat{ℍ}}_{\left[-k\right]}^{\left(\text{m}\right)}\beta^{\left(\text{m}\right)}-2\beta^{(\text{m})⊤}{\hat{\xi }}_{\left[-k\right]}^{\left(\text{m}\right)}$ provides a second order one–step approximation to the individual–level data loss function $2{\hat{𝓟}}_{{𝓘}_{-k}^{\left(\text{m}\right)}}f\left(X^{⊤}\beta^{\left(\text{m}\right)},Y\right)$ initializing with the local LASSO estimators. In contrast to Cai et al. (2019a), we introduce a cross–fitting procedure at each local DC to reduce fitting bias and this in turn relaxes their uniformly-bounded assumption on $X_{i}^{(\text{m})\text{T}}\beta^{\left(\text{m}\right)}$ for each i and m, i.e., Condition 4(i) of Cai et al. (2019a).

### 2.4 Step 2: Debiased group test statistics

We next derive group effect test statistics in Step 2 by constructing debiased estimators for $\beta_{0}^{\left(•\right)}$ and estimating their variances. In Step 2(a), we construct updated summary statistics

$${\tilde{\xi }}_{\left[k\right]}^{\left(\text{m}\right)}={\hat{𝓟}}_{{𝓘}_{k}^{\left(\text{m}\right)}}X_{{\tilde{\beta }}_{\left[-k\right]}^{\left(\text{m}\right)}}Y_{{\tilde{\beta }}_{\left[-k\right]}^{\left(\text{m}\right)}},{\tilde{ℍ}}_{\left[k\right]}^{\left(\text{m}\right)}={\hat{𝓟}}_{{𝓘}_{k}^{\left(\text{m}\right)}}X_{{\tilde{\beta }}_{\left[-k\right]}^{\left(\text{m}\right)}}X_{{\tilde{\beta }}_{\left[-k\right]}^{\left(\text{m}\right)}}^{⊤}\;\text{and}\;{\tilde{J}}_{\left[k\right]}^{\left(\text{m}\right)}={\hat{𝓟}}_{{𝓘}_{k}^{\left(\text{m}\right)}}XX^{⊤}{\left\{Y-\dot{\phi }\left(X^{⊤}{\tilde{\beta }}_{\left[-k\right]}^{\left(\text{m}\right)}\right)\right\}}^{2}$$

at the mth DC, for k∈[K]. These mK sets of summary statistics are then sent to the AC in Step 2(b) to be aggregated and debiased. Specifically, for each j∈𝓗 and k∈[K], we solve the group Dantzig selector type optimization problem:

(4) $${\hat{u}}_{j,[k]}^{\left(•\right)}=\underset{u^{\left(•\right)}}{\text{argmin}}\;\underset{m\in [M]}{\max}{\left\|u^{\left(\text{m}\right)}\right\|}_{1}\;\text{s.t.}\;{\left\|{\tilde{ℍ}}_{\left[k\right]}^{\left(•\right)}u^{\left(•\right)}-e_{j}^{\left(•\right)}\right\|}_{2,\infty }\leq \tau ,$$

to obtain a vector of projection directions for some tuning parameter τ, where ${\tilde{ℍ}}_{\left[k\right]}^{\left(•\right)}=\text{diag}\left\{{\tilde{ℍ}}_{\left[k\right]}^{\left(1\right)},\ldots ,{\tilde{ℍ}}_{\left[k\right]}^{\left(\text{M}\right)}\right\}$. Combining across the K splits, we construct the cross–fitted group debiased estimator for $\beta_{j}^{\left(\text{m}\right)}$ by ${\overset{˘}{\beta }}_{j}^{\left(\text{m}\right)}=K^{-1}\sum_{k=1}^{K}\left\{{\tilde{\beta }}_{j,[-k]}^{\left(\text{m}\right)}+{\hat{u}}_{j,[k]}^{(\text{m})\text{T}}\left({\tilde{\xi }}_{\left[k\right]}^{\left(\text{m}\right)}-{\tilde{ℍ}}_{\left[k\right]}^{\left(\text{m}\right)}{\tilde{\beta }}_{\left[-k\right]}^{\left(\text{m}\right)}\right)\right\}$.

In Section 3.2, we show that the distribution of $n_{m}^{1/2}\left({\overset{˘}{\beta }}_{j}^{\left(\text{m}\right)}-\beta_{0,j}\right)$) is approximately normal with mean 0 and variance ${\left(\sigma_{0,j}^{\left(\text{m}\right)}\right)}^{2}$, estimated by ${\left({\hat{\sigma }}_{j}^{\left(\text{m}\right)}\right)}^{2}=K^{-1}\sum_{k=1}^{K}{\hat{u}}_{j,[k]}^{(\text{m})⊤}{\tilde{J}}_{\left[k\right]}^{\left(\text{m}\right)}{\hat{u}}_{j,[k]}^{\left(\text{m}\right)}$. Finally, we test for the group effect of the j-th covariate across M studies based on the standardized sum of square type statistics

$${\overset{˘}{\zeta }}_{j}=\sum_{m=1}^{M}n_{m}{\left\{{\overset{˘}{\beta }}_{j}^{\left(\text{m}\right)}/{\hat{\sigma }}_{j}^{\left(\text{m}\right)}\right\}}^{2},\;\text{for}\;j\in 𝓗.$$

We show in Section 3.2 that, under mild regularity assumptions, ${\overset{˘}{\zeta }}_{j}$ is asymptotically chi-square distributed with degree of freedom M under the null. This result is crucial to ensure the error rate control for the downstream multiple testing procedure.

### 2.5 Step 3: Multiple testing

To construct an error rate controlled multiple testing procedure for

$$H_{0,j}:\beta_{0,j}=0\;\text{versus}\;H_{a,j}:\beta_{0,j}\neq 0,\;j\in 𝓗\subseteq \{2,\ldots ,p\},$$

we first take a normal quantile transformation of ${\overset{˘}{\zeta }}_{j}$, namely ${𝓝}_{j}={\bar{\text{Φ}}}^{-1}\left\{{\bar{F}}_{\chi_{M}^{2}}\left({\overset{˘}{\zeta }}_{j}\right)/2\right\}$, where Φ is the standard normal cumulative distribution function, $\bar{\text{Φ}}=1-\text{Φ}$, and ${\bar{F}}_{\chi_{M}^{2}}(\cdot )$ is the survival function of $\chi_{M}^{2}$. Based on the asymptotic $\chi_{M}^{2}$ distribution of ${\overset{˘}{\zeta }}_{j}$ as will be shown in Theorem 1, we present in the proof of Theorem 2 that ${𝓝}_{j}$ asymptotically has the same distribution as the absolute value of a standard normal random variable. Thus, to test a single hypothesis of $H_{0,j}:\beta_{0,j}=0$, we reject the the null at nominal level α>0 whenever ${\text{Ψ}}_{\alpha ,j}=1$, where ${\text{Ψ}}_{\alpha ,j}=I\left\{{𝓝}_{j}\geq {\bar{\text{Φ}}}^{-1}(\alpha /2)\right\}$.

However, for simultaneous inference across q hypotheses $\left\{H_{0,j},j\in 𝓗\right\}$, we shall further adjust the multiplicity of the tests as follows. For any threshold level t, let $R_{0}(t)=\sum_{j\in {𝓗}_{0}}I\left({𝓝}_{j}\geq t\right)$ and $R(t)=\sum_{j\in 𝓗}I\left({𝓝}_{j}\geq t\right)$ respectively denote the total number of false positives and the total number of rejections associated with t, where ${𝓗}_{0}=\{j\in 𝓗:\beta_{0,j}=0\}$. Then the FDP and FDR for a given t are respectively defined as

$$\text{FDP}(t)=\frac{R_{0}(t)}{R(t)\vee 1}\;\text{and}\;\text{FDR}(t)=\text{E}\{\text{FDR}(t)\}.$$

The smallest t such that FDP(t)≤α, namely $t_{0}=\inf\;\left\{0\leq t\leq {\left(2\log q\right)}^{1/2}:\;\text{FDP}(t)\leq \alpha \right\}$ would be a desirable threshold since it maximizes the power under the FDP control. However, since the null set is unknown, we estimate $R_{0}(t)$ by $2\bar{\text{Φ}}(t)\left|{𝓗}_{0}\right|$ and conservatively estimate $\left|{𝓗}_{0}\right|$ by q because of the model sparsity. We next calculate

(5) $$\hat{t}=\inf\left\{0\leq t\leq t_{q}:\frac{2q\bar{\text{Φ}}(t)}{R(t)\vee 1}\leq \alpha \right\}\;\text{where}\;t_{q}={\left(2\log q-2\log\log q\right)}^{\frac{1}{2}}$$

to approximate the ideal threshold $t_{0}$. If (5) does not exist, we set $\hat{t}={\left(2\log q\right)}^{1/2}$. Finally, we obtain the rejection set $\left\{j:{𝓝}_{j}\geq \hat{t},j\in 𝓗\right\}$ as the output of Algorithm 1. The theoretical analysis of the asymptotic error rates control of the proposed multiple testing procedure will be studied in Section 3.3.

#### Remark 1.

*Our testing approach is different from the BH procedure (Benjamini and Hochberg, 1995) in that, the latter obtains the rejection set* $\left\{j:{𝓝}_{j}\geq {\hat{t}}_{BH},j\in 𝓗\right\}$ *with* ${\hat{t}}_{BH}=\inf\{t\geq 0:2q\bar{\text{Φ}}(t)/\{R(t)\vee 1\}\leq \alpha \}$. *Note that, first, the range* $\left[0,t_{q}\right]$ *in our procedure is critical, because when* $t\geq {\left(2\log q-\log\log q\right)}^{\frac{1}{2}}$, $R_{0}(t)$ *is no longer consistently estimated by* $2q\bar{\text{Φ}}(t)$. *As a result, the BH may not able to control the FDP with positive probability. Second, in the proposed approach, if* $\hat{t}$ *is not attained in the range, it is crucial to threshold it at* ${\left(2\log q\right)}^{1/2}$, *instead of* $t_{q}$, *because the latter will cause too many false rejections, and as a result the FDR cannot be properly controlled*.

### 2.6 Tuning parameter selection

In this section, we detail data-driven procedures for selecting the tuning parameters $\eta =\left\{\lambda^{\left(•\right)}={\left(\lambda^{\left(1\right)},\ldots ,\lambda^{\left(\text{M}\right)}\right)}^{⊤},\lambda ,\tau \right\}$. Since our primary goal is to perform simultaneous testing, we follow a similar strategy as that of Xia et al. (2018a) and select tuning parameters to minimize $\ell_{2}$ distance between ${\hat{R}}_{0}(t)/\left\{2\left|{𝓗}_{0}\right|\bar{\text{Φ}}(t)\right\}$ and its expected value of 1, where ${\hat{R}}_{0}(t)$ is an estimate of $R_{0}(t)$ from the testing procedure. However, unlike Xia et al. (2018a), it is not feasible to tune η simultaneously due to DataSHIELD constraints. We instead tune $\lambda^{\left(•\right)}$, λ and τ sequentially as detailed below. Furthermore, based on the theoretical analyses of the optimal rates for η given in Section 3, we select η within a set of candidate values that are of the same order as their respective optimal rates.

First for $\lambda^{\left(•\right)}$ in Algorithm 1, we tune $\lambda^{\left(\text{m}\right)}$ via cross validation within the mth DC. Second, to select λ for the integrative estimation in (3), we minimize an approximated generalized information criterion that only involves derived data from M studies. Specifically, we choose λ as the minimizer of $\text{GIC}\left(\lambda ,{\tilde{\beta }}_{[-k],\lambda }^{\left(•\right)}\right)=\text{Dev}\left({\tilde{\beta }}_{[-k],\lambda }^{\left(•\right)}\right)+\gamma \text{DF}\left(\lambda ,{\tilde{\beta }}_{[-k],\lambda }^{\left(•\right)}\right)$, where γ is some pre-specified scaling parameter, ${\tilde{\beta }}_{[-k],\lambda }^{\left(\text{m}\right)}$ is the estimator obtained with λ,

$$\text{Dev}\;\left(\beta^{\left(•\right)}\right)={\left|{𝓘}_{-k}\right|}^{-1}\sum_{m=1}^{M}\left|{𝓘}_{-k}^{\left(\text{m}\right)}\right|\left(\beta^{(\text{m})\text{T}}{\hat{ℍ}}_{\left[-k\right]}^{\left(\text{m}\right)}\beta^{\left(\text{m}\right)}-2\beta^{\left(\text{m}\right)}{\hat{\xi }}_{\left[-k\right]}^{\left(\text{m}\right)}\right)\;\text{and}$$

$$\text{DF}\;\left(\lambda ,\beta^{\left(•\right)}\right)={\left[\partial_{\hat{𝓢}}^{2}\left\{\text{Dev}\;\left(\beta^{\left(•\right)}\right)+\lambda {\left\|\beta_{-1}^{\left(•\right)}\right\|}_{2,1}\right\}\right]}^{-1}\left[\partial_{\hat{𝓢}}^{2}\text{Dev}\;\left(\beta^{\left(•\right)}\right)\right],$$

are respectively the approximated deviance and degree of freedom measures, $\hat{𝓢}$ is the set of non-zero elements in $\beta^{\left(•\right)}$ and the operator $\partial_{\hat{𝓢}}^{2}$ denotes the second order partial derivative with respect to $\beta_{\hat{𝓢}}^{\left(•\right)}$. Common choices of γ include $2{\left|{𝓘}_{-k}\right|}^{-1}(\text{AIC})$, ${\left|{𝓘}_{-k}\right|}^{-1}\log\left|{𝓘}_{-k}\right|(\text{BIC})$, ${\left|{𝓘}_{-k}\right|}^{-1}\log\left|{𝓘}_{-k}\right|\log\log p$ (Wang et al., 2009, modified BIC) and $2{\left|{𝓘}_{-k}\right|}^{-1}\log\left|{𝓘}_{-k}\right|\log p$ (Foster and George, 1994, RIC). For numerical studies in Sections 4 and 5, we use BIC which appears to perform well across settings.

At the last step, we tune τ by minimizing an $\ell_{2}$ distance between ${\hat{R}}_{0,\text{null}}(t|\tau )/\{2q\bar{\text{Φ}}(t)\}$ and 1, where ${\hat{R}}_{0,\text{null}}(t|\tau )$ is an estimate of $R_{0}(t)$ with a given tuning parameter τ, and we replace ${𝓗}_{0}$ by q as in Xia et al. (2018a). Our construction of ${\hat{R}}_{0,\text{null}}(t|\tau )$ differs from that of Xia et al. (2018a) in that we estimate $R_{0}(t)$ under the complete null to better approximate the denominator of $2q\bar{\text{Φ}}(t)$. As detailed in Algorithm 2, we construct ${\overset{˘}{\beta }}_{j,\text{null}}^{\left(\text{m}\right)}$ as the difference between the estimator obtained with the first K/2 folds of data and the corresponding estimator obtained using the second K/2 folds of data, which is always centered around 0 rather than $\beta_{0j}^{\left(\text{m}\right)}$. Since the accuracy of ${\hat{R}}_{0,\text{null}}(t|\tau )$ for large t is most relevant to the error control, we construct the distance measure $\hat{d}(\tau )$ in Algorithm 2 focusing on t around ${\bar{\text{Φ}}}^{-1}\left[\bar{\text{Φ}}\left\{{\left(2\log q\right)}^{1/2}\right\}\iota \right]$ for some values of ι∈(0,1].

**Algorithm 2:** Selection of τ for multiple testing.

1.	For any given τ and each j∈𝓗, calculate ${\overset{˘}{\zeta }}_{j,\text{null}}(\tau )=\sum_{m=1}^{M}n_{m}{\left\{{\overset{˘}{\beta }}_{j,\text{null}}^{\left(\text{m}\right)}(\tau )/{\hat{\sigma }}_{j}^{\left(\text{m}\right)}\right\}}^{2}$ with
	${\overset{˘}{\beta }}_{j,\text{null}}^{\left(\text{m}\right)}(\tau )=K^{-1}\sum_{k=1}^{K}{\left(-1\right)}^{k>K/2}\left\{{\tilde{\beta }}_{j,[-k]}^{\left(\text{m}\right)}+{\hat{u}}_{j,[k]}^{(\text{m})\text{T}}(\tau )\left({\tilde{\xi }}_{\left[k\right]}^{\left(\text{m}\right)}-{\tilde{ℍ}}_{\left[k\right]}^{\left(\text{m}\right)}{\tilde{\beta }}_{\left[-k\right]}^{\left(\text{m}\right)}\right)\right\},$
	where ${\hat{u}}_{j,[k]}^{\left(•\right)}(\tau )$ is the debiasing projection direction obtained at tuning value τ.
2.	Define ${\hat{R}}_{0,\text{null}}(t|\tau )=\sum_{j\in 𝓗}I\left[{\bar{F}}_{\chi_{M}^{2}}\left\{{\overset{˘}{\zeta }}_{j,\text{null}}(\tau )\right\}\leq 2\bar{\text{Φ}}(t)\right]$ and a modified measure
	$\hat{d}(\tau )=\int_{0}^{1}{\left[{\hat{R}}_{0,\text{null}}\left\{{\bar{\text{Φ}}}^{-1}(x)|\tau \right\}/(2qx)-1\right]}^{2}d\hat{\omega }(x),$
	where $\hat{\omega }(x)=H^{-1}\sum_{h=1}^{H}I\left(\bar{\text{Φ}}\left\{{\left(2\log q\right)}^{1/2}\right\}h/H\leq x\right)$ and H>0 is some specified constant.

## 3. Theoretical Results

In this section, we present the asymptotic analysis results of the proposed method and compare it with alternative approaches.

### 3.1 Notation and assumptions

For any semi–positive definite matrix $A\in {\mathbb{R}}^{d\times d}$ and i,j∈[d], denote by $A_{ij}$ the ${\left(i,j\right)}^{\text{th}}$ element of A and $A_{j}$ its $j^{\text{th}}$ row, ${\text{Λ}}_{\min}(A)$ and ${\text{Λ}}_{\max}(A)$ the smallest and largest eigenvalue of A. Define the sub-gaussian norms of a random variable X and a d-dimensional random vector X, respectively by $∥X{∥}_{\psi_{2}}:=\sup_{q\geq 1}q^{-1/2}{\left(\text{E}|X{|}^{q}\right)}^{1/q}$ and $∥X{∥}_{\psi_{2}}:=\sup_{x\in S^{d-1}}{\left\|x^{⊤}X\right\|}_{\psi_{2}}$, where $S^{d-1}$ is the unit sphere in ${\mathbb{R}}^{d}$. For c>0 and a scalar or vector x, define $𝓑(x,c):=\left\{x^{\prime }:{\left\|x^{\prime }-x\right\|}_{1}\leq c\right\}$ as its $\ell_{1}$ neighbor with radius c. Denote by ${\text{Σ}}_{0}^{\left(\text{m}\right)}=𝓟(\text{m})XX^{⊤}$, ${ℍ}_{\beta }^{\left(\text{m}\right)}={𝓟}^{\left(\text{m}\right)}X_{\beta }X_{\beta }^{⊤},J_{\beta }^{\left(\text{m}\right)}={𝓟}^{\left(\text{m}\right)}XX^{⊤}{\left\{Y-\dot{\phi }\left(X^{⊤}\beta \right)\right\}}^{2}$ and $U_{\beta }^{\left(\text{m}\right)}={\left\{{ℍ}_{\beta }^{\left(\text{m}\right)}\right\}}^{-1}$. For simplicity, let ${ℍ}_{0}^{\left(\text{m}\right)}={ℍ}_{\beta_{0}^{\left(\text{m}\right)}}^{\left(\text{m}\right)}$, $J_{0}^{\left(\text{m}\right)}=J_{\beta_{0}^{\left(\text{m}\right)}}^{\left(\text{m}\right)}$ and denote by $u_{0,j}^{\left(\text{m}\right)}$ the $j^{\text{th}}$ row of $U_{\beta_{0}^{\left(\text{m}\right)}}^{\left(\text{m}\right)}$. In our following analysis, we assume that the cross–fitting folds $K^{\prime },K=O(1)$, $n_{m}≍N/M\equiv n$ for all m∈[M]. Here and in the sequel we use O(1) and $O_{\text{P}}(1)$ denote order 1. Next, we introduce assumptions for our theoretical results. For Assumption 4, we only require either 4(a) or 4(b) to hold.

#### Assumption 1

(Regular covariance). *(i) There exists absolute constant* $C_{\text{Λ}}>0$ *such that for all* m∈[M], $C_{\text{Λ}}^{-1}\leq {\text{Λ}}_{\min}\left({\text{Σ}}_{0}^{\left(\text{m}\right)}\right)\leq {\text{Λ}}_{\max}\left(\Sigma_{0}^{\left(\text{m}\right)}\right)\leq C_{\text{Λ}}$, $C_{\text{Λ}}^{-1}\leq {\text{Λ}}_{\min}\left({ℍ}_{0}^{\left(\text{m}\right)}\right)\leq {\text{Λ}}_{\max}\left({ℍ}_{0}^{\left(\text{m}\right)}\right)\leq C_{\text{Λ}}$ *and* $C_{\text{Λ}}^{-1}\leq {\text{Λ}}_{\min}\left(J_{0}^{\left(\text{m}\right)}\right)\leq {\text{Λ}}_{\max}\left(J_{0}^{\left(\text{m}\right)}\right)\leq C_{\text{Λ}}$. *(ii) There exist* $C_{\text{Ω}}>0$ *and* δ>0 *that for all* m∈[M] *and* $\beta \in 𝓑\left(\beta_{0}^{\left(\text{m}\right)},\delta \right)$, $\ell_{1}$ *norm of each row of* $U_{\beta }^{\left(\text{m}\right)}$ *is bounded by* $C_{\text{Ω}}$.

#### Assumption 2

(Smooth link function). *There exists a constant* $C_{L}>0$ *such that for all* $\theta ,\theta^{\prime }\in \mathbb{R}$, $\left|\ddot{\phi }(\theta )-\ddot{\phi }\left(\theta^{\prime }\right)\right|\leq C_{L}\left|\theta -\theta^{\prime }\right|$.

#### Assumption 3

(Sub-Gaussian residual). *For any* $x\in {\mathbb{R}}^{p},\epsilon_{i}^{\left(\text{m}\right)}$ *is conditional sub-Gaussian, i.e. there exists* κ(x) *such that* ${\left\|\epsilon_{i}^{\left(\text{m}\right)}\right\|}_{\psi_{2}}<\kappa (x)$ *given* $X_{i}^{\left(\text{m}\right)}=x$. *In addition, there exists some absolute constant* $C_{\epsilon }>0$ *such that, almost surely for* m=1,2…,M, $\kappa \left(X_{i}^{\left(\text{m}\right)}\right)\leq C_{\epsilon }$ *and* ${\ddot{\phi }}^{-1}\left(X_{i}^{(\text{m})⊤}\beta_{0}^{\left(\text{m}\right)}\right)\kappa^{2}\left(X_{i}^{\left(\text{m}\right)}\right)\leq C_{\epsilon }$.

#### Assumption 4(a)

(Sub-Gaussian design). $X_{i}^{\left(\text{m}\right)}$ *is sub-Gaussian, i.e. there exists some constant* κ>0 *that* ${\left\|X_{i}^{\left(\text{m}\right)}\right\|}_{\psi_{2}}<\kappa$.

#### Assumption 4(b)

(Bounded design). ${\left\|X_{i}^{\left(\text{m}\right)}\right\|}_{\infty }$ *is almost surely bounded by some absolute constant*.

#### Remark 2.

Assumptions 1 (i) and 4(a) (or 4(b)) are commonly used technical conditions in high dimensional inference in order to guarantee rate optimality of the regularized regression and debiasing approach (Negahban et al., 2012; Javanmard and Montanari, 2014). Assumptions 4(a) and 4(b) are typically unified by the sub-Gaussian design assumption (Negahban et al., 2012). In our analysis, they are separately studied, since ${\left\|X_{i}^{\left(\text{m}\right)}\right\|}_{\infty }$ *affects the bias rate, which leads to different sparsity assumptions under different design types. Similar conditions as our Assumption 1 (ii) were used in the context of high dimensional precision matrix estimation (Cai et al., 2011) and debiased inference (Chernozhukov et al., 2018; Caner and Kock, 2018; Belloni et al., 2018). Compared with their exact or approximate sparsity assumption imposed on the inverse Hessian, this* $\ell_{1}$ *boundness assumption is essentially less restrictive. As an important example in our analysis, logistics model satisfies the smoothness conditions for* ϕ(⋅) *presented by Assumption 2. As used in Lounici et al. (2011) and Huang and Zhang (2010), Assumption 3 regularizes the tail behavior of the residuals and is satisfied in many common settings like logistic model*.

### 3.2 Asymptotic properties of the debiased estimator

We next study the asymptotic properties of the group effect statistics ${\overset{˘}{\zeta }}_{j}$, j∈𝓗. We shall begin with some important prerequisite results on the convergence properties of ${\tilde{\beta }}_{\left[-k\right]}^{\left(•\right)}$ and the debiased estimators $\left\{{\overset{˘}{\beta }}_{j}^{\left(\text{m}\right)},j\in 𝓗,m\in [M]\right\}$ as detailed in Lemmas 1 and 2.

#### Lemma 1.

Under Assumptions 1-3, 4(a) *or* 4(b), *and that* $s=o\left\{n{\left(\log p\right)}^{-1}\right\}$, *there exist a sequence of the tuning parameters*

$$\lambda_{n}^{\left(\text{m}\right)}≍\frac{{\left(\log p\right)}^{\frac{1}{2}}}{n^{\frac{1}{2}}}\;\text{and}\;\lambda_{N}≍\frac{{\left(M+\log p\right)}^{\frac{1}{2}}}{n^{\frac{1}{2}}M}+\frac{sM^{-\frac{1}{2}}{\left(\log p+\log N\right)}^{a_{0}}\log p}{n},$$

*with* $a_{0}=1/2$ *under Assumption 4(a) and* $a_{0}=0$ *under Assumption 4(b), such that, for each* k∈[K], *the integrative estimator satisfies*

$${\left\|{\tilde{\beta }}_{\left[-k\right]}^{\left(•\right)}-\beta_{0}^{\left(•\right)}\right\|}_{2,1}=O_{\text{P}}\left(sM\lambda_{N}\right),\;\text{and}\;{\left\|{\tilde{\beta }}_{\left[-k\right]}^{\left(•\right)}-\beta_{0}^{\left(•\right)}\right\|}_{2}^{2}=O_{\text{P}}\left(sM^{2}\lambda_{N}^{2}\right).$$

#### Remark 3.

Lemma 1 *provides the estimation rates of the integrative estimator* ${\tilde{\beta }}_{\left[-k\right]}^{\left(•\right)}$. *In contrast to the ILMA method, the second term in the expression of* $\lambda_{N}$ *quantifies the additional noise incurred by using summary data under the DataSHIELD constraint. Similar results can be observed through debiasing truncation in distributed learning (Lee et al., 2017; Battey et al., 2018) or integrative estimation under DataSHIELD (Cai et al., 2019a). When* $s=o\left\{n^{1/2}{\left(\log p+\log N\right)}^{-a_{0}}{\left(M+\log p\right)}^{-1}{\left(\log p\right)}^{-1/2}\right\}$ *as assumed in Lemma 2, the above mentioned error term becomes negligible. The DSILT method allows for any degree of heterogeneity across sites with respect to both the magnitude and support of* $\beta_{0}^{\left(\text{m}\right)}$. *However, the cross-site similarity in the support determines the estimation rates as shown in Lemma 1 above. Specifically, the DSILT estimator for* $\beta^{\left(•\right)}$ *attains a rate-M improvement over the local methods (Lounici et al., 2011; Huang and Zhang, 2010, e.g.) if* $S≍S^{\left(\text{m}\right)}$ *and has the same rate as that of the local estimators if* $s≍\sum_{m=1}^{M}s^{\left(\text{m}\right)}$.

We next present the theoretical properties of the group debiased estimators.

#### Lemma 2.

*Under the same assumptions of Lemma 1 and assume that*

$$s=o\left\{\frac{n^{\frac{1}{2}}}{{\left(\log p+\log N\right)}^{a_{0}}(M+\log p){\left(\log p\right)}^{\frac{1}{2}}}\wedge \frac{n}{M^{4}{\left(\log p\right)}^{4}(M+\log p)}\right\},$$

*we have* ${\overset{˘}{\beta }}_{j}^{\left(\text{m}\right)}-\beta_{0,j}^{\left(\text{m}\right)}=V_{j}^{\left(\text{m}\right)}+{\text{Δ}}_{j}^{\left(\text{m}\right)}$ *with* $V_{j}^{\left(\text{m}\right)}=K^{-1}\sum_{k=1}^{K}{\hat{𝓟}}_{{𝓘}_{k}^{\left(\text{m}\right)}}u_{0,j}^{(\text{m})⊤}X\epsilon$ *converging to a normal random variable with mean* 0 *and variance* $n_{m}^{-1}{\left(\sigma_{0,j}^{\left(\text{m}\right)}\right)}^{2}$, *where* ${\left(\sigma_{0,j}^{\left(\text{m}\right)}\right)}^{2}=u_{0,j}^{(\text{m})\text{T}}J_{0}^{\left(\text{m}\right)}u_{0,j}^{\left(\text{m}\right)}$. *In addition, there exists* $\tau ≍{\left(M+\log p\right)}^{1/2}n^{-1/2}$ *such that, simultaneously for all* j∈𝓗, *the bias term* ${\text{Δ}}_{j}^{\left(\text{m}\right)}$ *and the variance estimator* ${\left({\hat{\sigma }}_{j}^{\left(\text{m}\right)}\right)}^{2}$ *satisfy that*

$$\left|{\text{Δ}}_{j}^{\left(\text{m}\right)}\right|\leq \sum_{m=1}^{M}\left|{\text{Δ}}_{j}^{\left(\text{m}\right)}\right|=o_{\text{P}}\left\{{\left(n\log p\right)}^{-\frac{1}{2}}\right\}\;\text{and}\;\left|{\left({\hat{\sigma }}_{j}^{\left(\text{m}\right)}\right)}^{2}-{\left(\sigma_{0,j}^{\left(\text{m}\right)}\right)}^{2}\right|=o_{\text{P}}\left\{{\left(\log p\right)}^{-1}\right\}.$$

#### Remark 4.

*The sparsity assumption in Lemma 2 is weaker than the existing debiased estimators for M–estimation where s is only allowed to diverge in a rate dominated by* $N^{\frac{1}{3}}$ *(Janková and Van De Geer, 2016; Belloni et al., 2018; Caner and Kock, 2018). This is benefited by the cross–fitting technique, through which we can get rid of the dependence on the convergence rate of* ${\left\|u_{0,j}^{\left(\text{m}\right)}-{\hat{u}}_{j,[k]}^{\left(\text{m}\right)}\right\|}_{1}$.

Finally, we establish in Theorem 1 the main result of this section regarding to the asymptotic distribution of the group test statistic ${\overset{˘}{\zeta }}_{j}$ under the null.

#### Theorem 1.

*Under all assumptions in Lemma 2, simultaneously for all* $j\in {𝓗}_{0}$, *we have* ${\overset{˘}{\zeta }}_{j}=S_{j}+o_{\text{P}}(1)$, *where* $S_{j}=\sum_{m=1}^{M}n_{m}{\left[V_{j}^{\left(\text{m}\right)}/\sigma_{0,j}^{\left(\text{m}\right)}\right]}^{2}$. *Furthermore, if* M≤Clogp *and* $\log p=o\left(n^{1/C^{\prime }}\right)$ *for some constants* C>0 *and* $C^{\prime }>6$, *we have*

$$\underset{t}{\sup}\left|\text{P}\left(S_{j}\leq t\right)-\text{P}\left(\chi_{M}^{2}\leq t\right)\right|\to 0,\;\text{as}\;n,p\to \infty .$$

The above theorem shows that, the group effect test statistics ${\overset{˘}{\zeta }}_{j}$ is asymptotically chi-squared distributed under the null and its bias is uniformly negligible for $j\in {𝓗}_{0}$.

### 3.3 False discovery control

We establish theoretical guarantees for the error rate control of the multiple testing procedure described in Section 2.5 in the following two theorems.

#### Theorem 2.

*Assume that* $q_{0}=\left|{𝓗}_{0}\right|≍q$. *Then under all assumptions in Lemma 2 with* $\log p=o\left(n^{1/10}\right)$ *and* M=O(logp), *we have*

$$\underset{(N,p)\to \infty }{\text{lim sup}}\text{FDR}(\hat{t})\leq \alpha ,\;and\;\underset{(N,p)\to \infty }{\lim}P\{\text{FDP}(\hat{t})\leq \alpha +\epsilon \}=1\;for\;any\;\epsilon >0.$$

#### Remark 5.

*Assumption 1 (i) ensures that most of the group estimates* $\left\{{\overset{˘}{\zeta }}_{j},j\in {𝓗}_{0}\right\}$ *are not highly correlated with each other. Thus the the variance of* ${\hat{R}}_{0}(t)$ *can be appropriately controlled, which in turn guarantees the control of FDP. It is possible to further relax the condition* $\log p=o\left(n^{1/10}\right)$ *to* $\log p=o\left(n^{\zeta }\right)$ *for some* 0<ζ<3/23, *See, for example, Liu and Shao (2014) and Belloni et al. (2018), where they used moderate deviation technique to have tighter truncations and normal approximations for t-statistics. Because we used chi-squared type test statistics with growing M, the technical details on moderate deviation are much more involved and warrant future research*.

As described in Section 2.5, if $\hat{t}$ in equation (5) is not attained in the range $[0,(2\log q-2\log\log q{)}^{1/2}]$, then it is thresholded at ${\left(2\log q\right)}^{1/2}$. The following theorem states a weak condition to ensure the existence of $\hat{t}$ in such range. As a result, the FDP and FDR will converge to the pre-specified level α asymptotically.

#### Theorem 3.

*Let* ${𝓢}_{\rho }=\left\{j\in 𝓗:\sum_{m=1}^{M}n_{m}{\left[\beta_{0,j}^{\left(\text{m}\right)}\right]}^{2}\geq {\left(\log q\right)}^{1+\rho }\right\}$. *Suppose for some* ρ>0 *and some* δ>0, $\left|{𝓢}_{\rho }\right|\geq \left\{1/\left(\pi^{1/2}\alpha \right)+\delta \right\}{\left(\log q\right)}^{1/2}$. *Then under the same conditions as in Theorem 2, we have, as* (N,p)→∞,

$$\frac{\text{FDR}(\hat{t})}{\alpha q_{0}/q}\to 1,\;\frac{\text{FDP}(\hat{t})}{\alpha q_{0}/q}\to 1\;in\;probability.$$

In the above theorem, the condition on ${𝓢}_{\rho }$ only requires very few covariates to have the signal sum of squares across the studies $\sum_{m=1}^{M}{\left[\beta_{0,j}^{\left(\text{m}\right)}\right]}^{2}$ exceeding the rate ${\left(\log q\right)}^{1+\rho }/n_{m}$ for some ρ>0, and is thus a very mild assumption.

### 3.4 Comparison with alternative approaches

To study the advantage of our testing approach and the impact of the DataSHIELD constraint, we next compare the proposed DSILT method to a One–shot approach and the ILMA approach, as described in Algorithms 3 and 4, through a theoretical perspective. The One–shot approach in Algorithm 3 is inspired by existing literature in distributed learning (Lee et al., 2017; Battey et al., 2018, e.g.), and is a natural extension of existing methods to the problem of multiple testing under the DataSHIELD constraint. The debiasing step of the One–shot approach is performed locally as in the existing literature.

Following similar proofs of Lemma 2 and Theorems 2 and 3, the One–shot, ILMA, and DSILT can attain the same error rate control results under the sparsity assumptions of

$$s=o\left(\gamma_{1}\wedge \gamma_{2}\right),\;(\text{One-shot)}\;\text{and}\;s=o\left\{\left(\gamma_{1}M\right)\wedge \gamma_{2}\right\}\;\text{(ILMA/DSILT),}$$

**Algorithm 3:** One–shot approach.

1.	At each DC, obtain the cross–fitted debiased estimator by solving a Dantzig selector problem locally, where $\beta^{\left(\text{m}\right)}$ is estimated by local LASSO.
2.	Send the debiased estimators to the AC and obtain the group statistics.
3.	Perform multiple testing procedure as described in Section 2.5.

**Algorithm 4:** Individual–level meta–analysis (ILMA).

1.	Integrate all individual–level data at the AC.
2.	Construct the cross–fitted debiased estimator by (4) using individual–level integrative estimator analog to (3), and then obtain the overall effect statistics.
3.	Perform multiple testing procedure in Section 2.5.

where under the high dimensional regime of logn=O(logp) and the assumptions of M=O(logp) and $\log p=o\left(n^{1/10}\right)$ as required in Theorems 2 and 3,

$$\gamma_{1}=\frac{n^{\frac{1}{2}}}{M{\left(\log p+\log n\right)}^{a_{0}}{\left(\log p\right)}^{\frac{3}{2}}}≍\frac{n^{\frac{1}{2}}}{M{\left(\log p\right)}^{a_{0}+\frac{3}{2}}},\;\gamma_{2}=\frac{n}{M^{4}{\left(\log p\right)}^{5}},$$

and $a_{0}=1/2$ for sub-Gaussian design and $a_{0}=0$ for bounded design as in Lemma 1. If additionally $M=o\left\{n^{1/6}{\left(\log p\right)}^{a_{0}/3-7/6}\right\}$ which directly implies $\gamma_{1}=o\left(\gamma_{2}\right)$, then the respective sparsity conditions for One–shot and ILMA/DSILT reduce to $s=o\left(\gamma_{1}\right)$ and $s=o\left\{\left(\gamma_{1}M\right)\wedge \gamma_{2}\right\}$. Hence, when M grows with n and p at a slower rate of $M=o\left\{n^{1/6}{\left(\log p\right)}^{a_{0}/3-7/6}\right\}$, we have $\gamma_{1}=o\left\{\left(\gamma_{1}M\right)\wedge \gamma_{2}\right\}$, which implies that the ILMA and DSILT methods require strictly weaker sparsity assumption than the One–shot approach. On the other hand, if $M=o\left(n^{1/6}{\left(\log p\right)}^{a_{0}/3-7/6}\right)$ is not satisfied, then the rate $\gamma_{2}$ dominates the rate of s and the three methods share the same sparsity condition $s=o\left(\gamma_{2}\right)$. Besides the sparsity condition comparisons in terms of the validity of tests, we learn from Cai et al. (2019a) that the estimation error rate of our integrative sparse regression in Step 1 is equivalent to the idealized method with all raw data and is smaller than the local estimator. Hence, we anticipate the power gain of the DSILT over the One–shot approach in finite-sample studies as the former uses more accurate estimator than the latter to derive statistics for debiasing. This advantage is also verified in our simulation studies in Section 4. Moreover, it is possible to follow the debiasing strategies proposed in Zhu et al. (2018) and Dukes and Vansteelandt (2019) that adapts to model sparsity, and construct a corresponding DSILT procedure with additional theoretical power gain compared with the One-shot method.

#### Remark 6.

*Our DSILT approach involves transferring data twice from the DCs to the AC and once from the AC to the DCs, which requires more communication efforts compared to the One–shot approach. The additional communication gains lower bias rate than the One–shot approach while only requiring the same sparsity assumption as the ILMA method as discussed above. Under its sparsity condition, each method is able to draw inference that is asymptotically valid and has the same power as the ideal case when one uses the true parameters in construction of the group test statistics. This further implies that to construct a powerful and valid multiple testing procedure, there is no necessity to adopt further sequential communications between the DCs and the AC as in the distributed methods of Li et al. (2016) and Wang et al. (2017)*.

## 4. Simulation Study

We evaluate the empirical performance of the DSILT procedure and compare it with the One–shot and the ILMA methods. Throughout, we let M=5, $n_{m}=500$, and vary p from 500 to 1000. For each setting, we perform 200 replications and set the number of sample splitting folds K=2, $K^{\prime }=5$ and false discovery level α=0.1. The tuning strategies described in Section 2.6 are employed with H=10.

The covariate X of each study is generated from either the (i) Gaussian auto–regressive (AR) model of order 1 and correlation coefficient 0.5; or (ii) Hidden Markov model (HMM) with binary hidden variables and binary observed variables with the transition probability and the emission probability both set as 0.2. We choose $\left\{\beta_{0}^{\left(\text{m}\right)}\right\}$ to be heterogeneous in magnitude across studies but to share the same support with

$$\beta_{0}^{\left(\text{m}\right)}=\mu {\left\{\left(\nu_{1}^{\left(\text{m}\right)}+1\right)\psi_{1},\left(\nu_{2}^{\left(\text{m}\right)}+1\right)\psi_{2},\ldots ,\left(\nu_{s}^{\left(\text{m}\right)}+1\right)\psi_{s},0_{p-s}\right\}}^{⊤}$$

where the sparsity level s is set to be 10 or 50, and $\left\{\psi_{1},\ldots ,\psi_{s}\right\}$ are independently drawn from {−1,1} with equal probability and are shared across studies, while the local signal strength $\nu_{j}^{\left(\text{m}\right)}$’s vary across studies and are drawn independently from $\text{N}\left\{0,{\left(\mu /2\right)}^{2}\right\}$. To ensure the procedures have reasonable power magnitudes for comparison, we set the overall signal strength μ to be in the range of [0.21,0.42] for s=10, mimicking a sparse and strong signal setting; and [0.14,0.35] for s=50, mimicking a dense and weak signal setting. We then generate binary responses $Y^{\left(\text{m}\right)}$ from $\text{logit}P\left(Y^{\left(\text{m}\right)}=1|X^{\left(\text{m}\right)}\right)=\beta_{0}^{(\text{m})\text{T}}X^{\left(\text{m}\right)}$.

**Figure 1:**
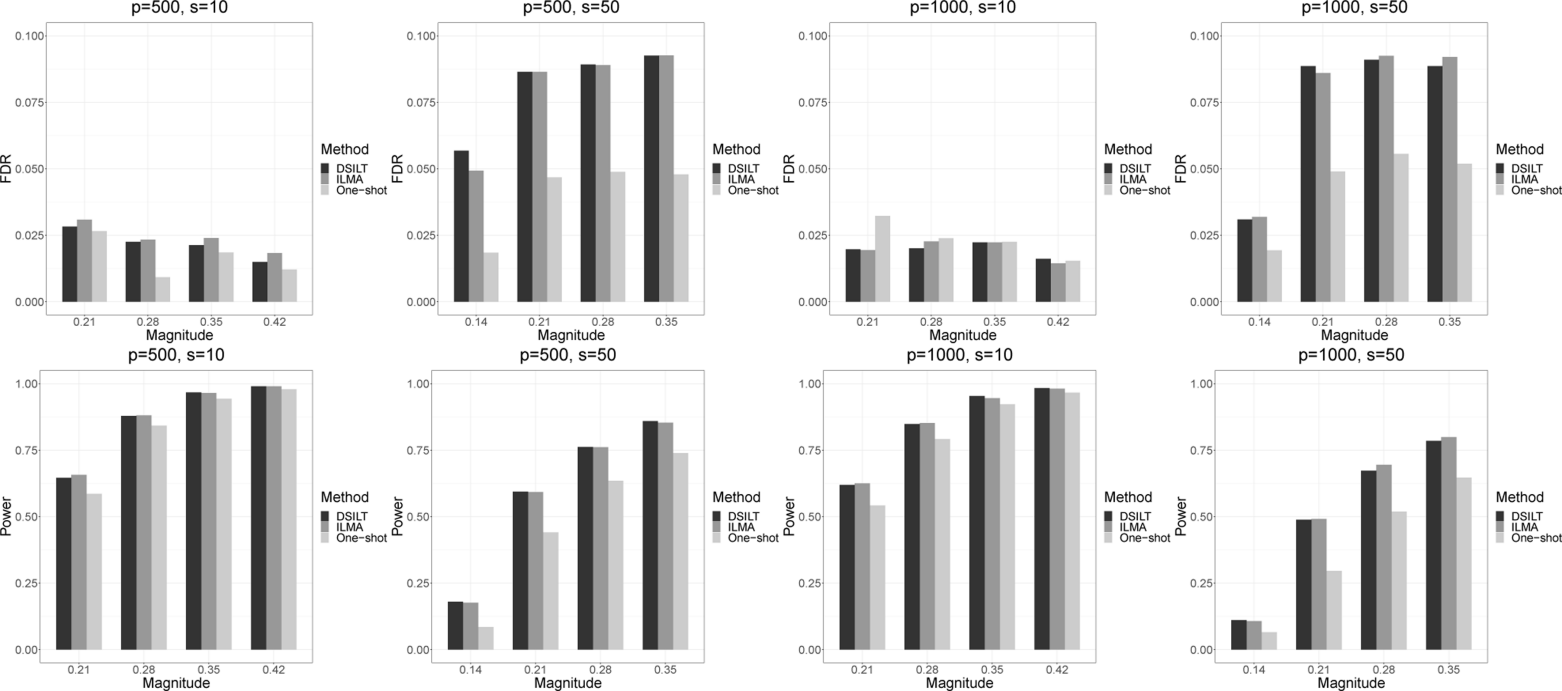
The empirical FDR and power of our DSILT method, the One–shot approach and the ILMA method under the Gaussian design, with α=0.1. The horizontal axis represents the overall signal magnitude μ.

In Figure 1, we report the empirical FDR and power of the three methods with varying p, s, and μ under the Gaussian design. Results for the HMM design have almost the same pattern and are included in the Supplementary Material. Across all settings, DSILT achieves almost the same performance as the ideal ILMA in both error rate control and power. All the methods successfully control the desired FDR at α=0.1. When s=10 or the signal strength μ is weak, all the methods have conservative error rates compared to the nominal level. While for s=50 with relatively strong signal, our method and the ideal ILMA become close to the exact error rate control empirically. This is consistent with Theorem 3 that if the number of relatively strong signals is large enough, our method tends to achieve exact FDR control. In contrast, the One–shot method fails to borrow information across the studies, and hence requires stronger signal magnitude to achieve exact FDR control. As a result, we observe consistently conservative empirical error rates for the One–shot approach.

In terms of the empirical power, the difference between DSILT and ILMA is less than 1% in all cases. This indicates that the proposed DSILT can accommodates the DataSHIELD constraint at almost no cost in power compared to ideal method. This is consistent with our theoretical result in Section 3.4 that the two methods require the same sparsity assumption for simultaneous inference. Furthermore, the DSILT and ILMA methods dominate the One–shot strategy in terms of statistical power. Under every single scenario, the power of the former two methods is around 15% higher than that of the One–shot approach in the dense case, i.e., s=50, and 6% higher in the sparse case, i.e, s=10. By developing testing procedures using integrative analysis rather than local estimations, both DSILT and ILMA methods use the group sparsity structure of the model parameters $\beta^{\left(•\right)}$ more adequately than the One–shot approach, which leads to the superior power performance of these two methods. The power advantage is more pronounced as the sparsity level *s* grows from 10 to 50. This is due to the fact that, to achieve the same result, the One–shot approach requires a stronger sparsity assumption than the other two methods, and is thus much more easily impacted by the growth of *s*. In comparison, the performance of our method and the ILMA method is less sensitive to sparsity growth because the integrative estimator employed in these two methods is more stable than the local estimator under the dense scenario.

## 5. Real Example

Statins are the most widely prescribed drug for lowering low–density lipoprotein (LDL) and the risk of cardiovascular disease (CVD), with over a quarter of adults 45 years or older receiving the drug in the United States. Statins lower LDL by inhibiting 3-hydroxy-3-methylglutaryl-coenzyme A reductase (HMGCR) (Nissen et al., 2005). The treatment effect of statins can also be causally inferred based on the effect of the HMGCR variant *rs17238484* – patients carrying the *rs17238484*-G allele have profiles similar to individuals receiving statin, with lower LDL and lower risk of CVD (Swerdlow et al., 2015). While the benefit of statins have been consistently observed, they are not without risk. There has been increasing evidence that statins increase the risk of type II diabetes (T2D) (Rajpathak et al., 2009; Carter et al., 2013). Swerdlow et al. (2015) demonstrated via both meta analysis of clinical trials and genetic analysis of the *rs17238484* variant that statins are associated with a slight increase of T2D risk. However, the adverse effect of statins on T2D risk appears to differ substantially depending on the number of T2D risk factors patients have prior to receiving the statin, with adverse risk higher among patients with more risk factors (Waters et al., 2013).

To investigate potential genetic determinants of statin treatment effect heterogeneity, we studied interactive effects of the *rs17238484* variant and 256 SNPs associated with T2D, LDL, high–density lipoprotein (HDL) cholesterol, and the coronary artery disease (CAD) gene which plays a central role in obesity and insulin sensitivity (Kozak and Anunciado-Koza, 2009; Rodrigues et al., 2013). A significant interaction between SNP j and the statin variant *rs17238484* would indicate that SNP j modifies the effect of statin. Since the LDL, CAD and T2D risk profiles differ greatly between different racial groups and between male and female, we focus the analysis on the black sub-population and fit separate models for female and male subgroups.

To efficiently identify genetic risk factors that significantly interact with *rs17238484*, we performed an integrative analysis of data from 3 different studies, including the Million Vetern Project (MVP) from the Veteran Health Administration (Gaziano et al., 2016), Partners Healthcare Biobank (PHB) and the UK Biobank (UKB). Within each study, we have both a male subgroup indexed by subscript m, and a female subgroup indexed by subscript f, leading to M=6 datasets denoted by ${\text{MVP}}_{\text{F}}$, ${\text{MVP}}_{\text{M}}$, ${\text{PHB}}_{\text{F}}$, ${\text{PHB}}_{\text{M}}$, ${\text{UKB}}_{\text{F}}$ and ${\text{UKB}}_{\text{M}}$. Since T2D prevalence within the datasets varies greatly from 0.05% to 0.15%, we performed a case control sampling with 1:1 matching so each dataset has equal numbers of T2D cases and controls. Since MVP has a substantially larger number of male T2D cases than all other studies, we down sampled its cases to match the number of female cases in MVP so that the signals are not dominated by the male population. This leads to sample sizes of 216, 392, 606, 822, 3120 and 3120 at ${\text{PHB}}_{\text{M}}$, ${\text{PHB}}_{\text{F}}$, ${\text{UKB}}_{\text{M}}$, ${\text{UKB}}_{\text{F}}$, ${\text{MVP}}_{\text{M}}$ and ${\text{MVP}}_{\text{F}}$, respectively. The covariate vector $X={\left(X_{\text{main}}^{⊤},X_{\text{int}}^{⊤}\right)}^{⊤}$ is of dimension p=516, where $X_{\text{main}}$ consists of the main effects of *rs17238484*, age and the aforementioned 256 SNPs, and $X_{\text{int}}$ consists of the interactions between *rs17238484* and age, as well as each of the 256 SNPs. All SNPs are encoded such that the higher value is associated with higher risk of T2D. We implemented the proposed testing method along with the One–shot approach as a benchmark to perform multiple testing of q=256 coefficients corresponding to the interaction terms in $X_{\text{int}}$ at nominal level of α=0.1 with the model chosen as logistics regression and the sample splitting folds K=2 and $K^{\prime }=5$.

**Figure 2:**
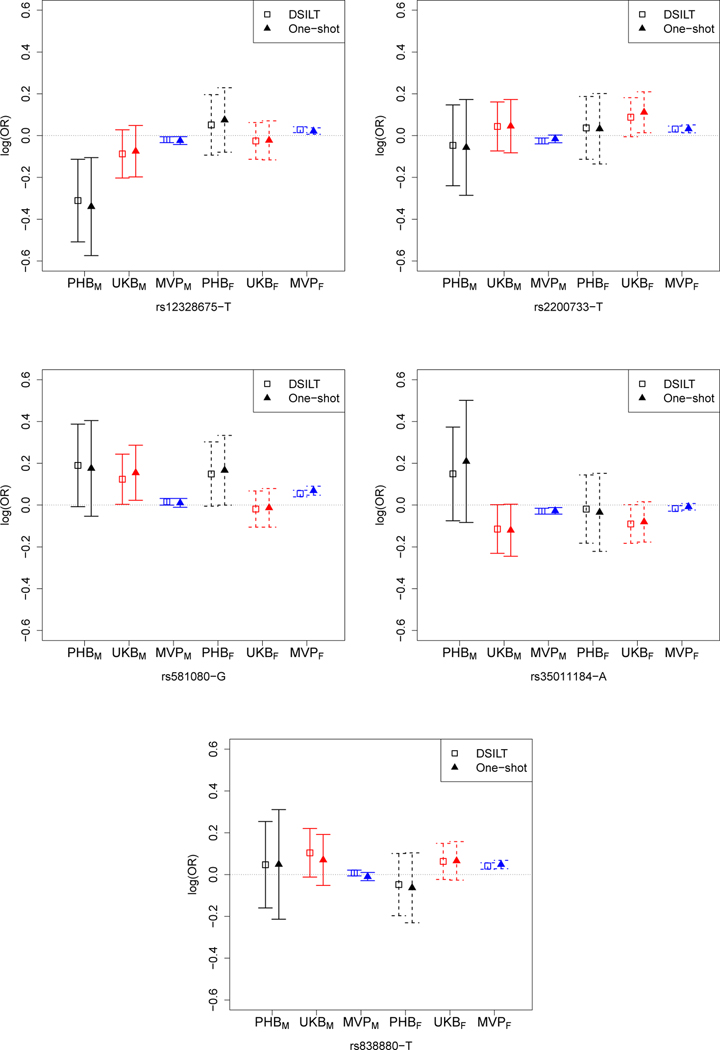
Debiased estimates of the log odds ratios and their 90% confidence intervals in each local site for the interaction effects between *rs17238484*-G and the 5 SNPs detected by DSILT, obtained respectively based on the One–shot and the DSILT approaches.

As shown in Table 1, our method identifies 5 SNPs significantly interacting with the statin SNP while the One–shot approach detects only 3 SNPs, all of which belong to the set of SNPs identified by our method. The presence of non-zero interactive effects demonstrates that the adverse effect of statin SNP *rs17238484*-G on the risk of T2D can differ significantly among patients with different levels of genetic predisposition to T2D. In Figure 2, we also present 90% confidence intervals obtained within each dataset for the interactive effects between *rs17238484*-G and each of these 5 detected SNPs. The SNP *rs581080*-G in the TTC39B gene has the strongest interactive effect with the statin SNP and has all interactive effects estimated as positive for most studies, suggesting that the adverse effect of statin is generally higher for patients with this mutation compared to those without. Interestingly, a previous report finds that a SNP in the TTC39B gene is associated with statin induced response to LDL particle number (Chu et al., 2015), suggesting that the effect of statin can be modulated by the *rs581080*-G SNP.

Results shown in Figure 2 also suggest some gender differences in the interactive effects. For example, the adverse effect of the statin is lower for female patients carrying the *rs12328675*-T allele compare to female patients without the allele. On the other hand, the effect of the statin appear to be higher for male patients with the *rs12328675*-T allele compared to those without genetic variants associated with a various of phenotypes related to T2D. The variation in the effect sizes across different data sources illustrates that it is necessary to properly account for heterogeneity of β in the modeling procedure. Comparing the lengths of confidence intervals obtained based on the One–shot approach to those from the proposed method, we find that the DSILT approach generally yields shorter confidence intervals, which translates to higher power in signal detection. It is important to note that since MVP has much larger sample sizes, the width of the confidence intervals from MVP are much smaller than those of UKB and PHB. However, the effect sizes obtained from MVP also tend to be much smaller in magnitude and consequently, using MVP alone would only detect 2 of the 5 SNPs by multiple testing with level 0.1. This demonstrates the utility of the integrative testing involving M=6 data sources.

## 6. Discussion

In this paper, we propose a DSILT method for simultaneous inference of high dimensional covariate effects in the presence of between-study heterogeneity under the DataSHIELD framework. The proposed method is able to properly control the FDR and FDP in theory asymptotically, and is shown to have similar performance as the ideal ILMA method and to outperform the One–shot approach in terms of the required assumptions and the statistical power for multiple testing. Our method allows most distributional properties of the data ${𝓓}^{\left(\text{m}\right)}$ to differ across the M sites, such as the marginal distribution of $X^{\left(\text{m}\right)}$, the conditional variance of $Y^{\left(\text{m}\right)}$ given $X^{\left(\text{m}\right)}$, and the magnitude of each $\beta_{j}^{\left(\text{m}\right)}$. The support ${𝓢}^{\left(\text{m}\right)}$ is also allowed to vary across the sites as well, but the DSILT method is more powerful when ${𝓢}^{\left(1\right)},\ldots ,{𝓢}^{\left(\text{M}\right)}$ are more similar to each other. We demonstrate that the sparsity assumptions of the proposed method are equivalent to those for the ideal method but strictly weaker than those for the One–shot approach. As the price to pay, our method requires one more round of data transference between the AC and the DCs than the One–shot approach. Meanwhile, the sparsity condition equivalence between the proposed method and ILMA method implies that there is no need to include in our method further rounds of communications or adopt iterative procedures as in Li et al. (2016) and Wang et al. (2017), which saves a great deal of human effort in practice.

The proposed approach also adds technical contributions to existing literature in several aspects. First, our debiasing formulation helps to get rid of the group structure assumption on the covariates $X^{\left(\text{m}\right)}$ at different distributed sites. Such an assumption is not satisfied in our real data setting, but is unavoidable if one uses the node-wise group LASSO (Mitra et al., 2016) or group structured inverse regression (Xia et al., 2018a) for debiasing. Second, compared with the existing work on joint testing of high dimensional linear models (Xia et al., 2018a), our method considers model heterogeneity and allows the number of studies M to diverge under the data sharing constraint, resulting in substantial technical difficulties in characterizing the asymptotic distribution of our proposed test statistics ${\overset{˘}{\zeta }}_{j}$ and their correlation structures for simultaneous inference.

We next discuss the limitation and possible extension of the current work. First, the proposed procedure requires transferring of Hessian matrix with $O\left(p^{2}\right)$ complexity from each DC to the AC. To the best of our knowledge, there is no natural way to reduce the order of complexity for the group debiasing step, i.e., Step 2, as introduced in Section 2.4. Nevertheless, it is worthwhile to remark that, for the integrative estimation step, i.e., Step 1, the communication complexity can be reduced to O(p) only, by first transferring the locally debiased LASSO estimators from each DC to the AC and then integrating the debiased estimators with a group structured truncation procedure (Lee et al., 2017; Battey et al., 2018, e.g.) to obtain an integrative estimator with the same error rate as ${\tilde{\beta }}_{\left[-k\right]}^{\left(•\right)}$. However, such a procedure requires greater efforts in deriving the data at each DC, which is not easily accomplished in some situations such as in our real example. Second, we assume q=|𝓗|≍p in the current paper as we have q=p/2 in the real example of Section 5. We can further extend our results to the cases when q grows slower than p. In such scenarios, the error rate control results in Theorems 2 and 3 still hold. Meanwhile, the model sparsity assumptions and the conditions on p and N can be further relaxed because we have fewer number of hypotheses to test in total and as a result the error rate tolerance for an individual test $H_{0,j}$ can be weakened. Third, for the limiting null distribution of the test statistics ${\overset{˘}{\zeta }}_{j}$ and the subsequent simultaneous error rate control, we require M=O(logp) and $\log p=o\left(n^{1/10}\right)$. Such an assumption is naturally satisfied in many situations as in our real example. However, when the collaboration is of a larger scale, say M≫logp or $M>n_{m}$, developing an adaptive and powerful overall effect testing procedure (such as the $\ell_{\infty }$–type test statistics), particularly under DataSHIELD constraints, warrants future research. Fourth, the sub-Gaussian residual Assumption 3 in our theoretical analysis does not hold for Poisson or negatively binomial response. Inspired by existing work (Jia et al., 2019; Xie and Xiao, 2020, e.g.), our framework can be potentially generalized to accommodate more types of outcome models. Last, our method may be modified by perturbing the weighted covariates $X_{\hat{\beta }}^{\left(\text{m}\right)}$ and response $Y_{\hat{\beta }}^{\left(\text{m}\right)}$, and transferring the summary statistics derived from the perturbed data. Designing such a method with more convincing privacy guarantees, as well as similar estimation and testing performance as in our current framework warrants future research.

## Supplementary Material

Supplement Materials

## Figures and Tables

**Table 1:** SNPs identified by DSILT to interact with the statin genetic variants *rs17238484*-G on the risk for T2D. The second column presents the name of the gene where the SNP locates. The third column presents the minor allele frequency (MAF) of each SNP averaged over the three sites. The last three columns respectively present the p–values obtained using One–shot approach with all the M=6 studies, One–shot with solely the datasets ${\text{MVP}}_{f}$ and ${\text{MVP}}_{m}$ and the proposed method with all the M=6 studies. The p–values shown in black fonts represent the SNPs selected by each method.

SNP	Gene	MAF	One–shot	MVP–only	DSILT
*rs12328675*-T	COBLL1	0.13	**1.1×10^−3^**	2.3 × 10^−3^	**6.0×10^−4^**
*rs2200733*-T	LOC729065	0.18	3.7 × 10^−2^	5.7 × 10^−3^	**6.2×10^−4^**
*rs581080*-G	TTC39B	0.22	**3.6×10^−6^**	**1.1×10^−6^**	**2.6×10^−6^**
*rs35011184*-A	TCF7L2	0.22	1.9 × 10^−2^	5.2 × 10^−2^	**8.6×10^−4^**
*rs838880*-T	SCARB1	0.36	**6.7×10^−4^**	**6.0×10^−5^**	**6.2×10^−4^**

## Footnotes

1 . Though a Poisson distribution does not satisfy the required sub-Gaussian residual Assumption 3, the counts of EHR diagnostic codes are usually less heavy-tailed than Poisson and are accommodated by our analysis.
