# Supplementary material for: Integrative High Dimensional Multiple Testing with Heterogeneity under Data Sharing Constraints
Source: J Mach Learn Res. Author manuscript; Available in PMC 2023 Jul 7. (PMC10327421)
Supplement: Supplement Materials [file NIHMS1912652-supplement-Supplement_Materials.pdf]

Supplement to "Integrative High Dimensional Multiple Testing with Heterogeneity under Data Sharing Constraints"

Molei Liu, Yin Xia[1], Kelly Cho and Tianxi Cai

In this supplement we provide proofs for the theoretical results in the paper, collect technical lemmas that are used in the proofs and present additional simulation results.

S1. PROOF

In this section, we present proofs of the theoretical results in the paper. Technical lemmas, Lemmas S1-S6, used in the proofs will be collected in Section S2.

Throughout, for a vector or matrix $\mathbf{A}(t) = [A_{ij}(t)]$, a function of the scalar $t \in [0,1]$, define $\int_0^1 \mathbf{A}(t)dt = [\int_0^1 A_{ij}(t)dt]$. For any matrix $\mathbf{A} = [A_{ij}]$, $\|\mathbf{A}\|_{\max} = \max_{ij}|A_{ij}|$. Additionally, we define the Restricted Eigenvalue Condition ($\mathscr{C}_{\mathsf{RE}}$) for data from M studies as follows.

Definition S1. Restricted Eigenvalue Condition ($\mathscr{C}_{\mathsf{RE}}$): *Let $\mathcal{C}(t,\mathcal{S}) = \{\boldsymbol{u}^{(\bullet)} \in \mathbb{R}^{p\times M} : \|\boldsymbol{u}^{(\bullet)}_{\mathcal{S}^c}\|_{2,1} \le t\|\boldsymbol{u}^{(\bullet)}_{\mathcal{S}}\|_{2,1}\}$. The covariance matrices $\boldsymbol{\Sigma} = \mathsf{diag}\{\boldsymbol{\Sigma}^{(1)}, \boldsymbol{\Sigma}^{(2)}, \ldots, \boldsymbol{\Sigma}^{(\mathsf{m})}\}$ and set $\mathcal{S} \subseteq [p]$ satisfy Restricted Eigenvalue Condition with some constant t: if there exists $\phi_0(t,\mathcal{S},\boldsymbol{\Sigma})$, for any $\boldsymbol{\delta}^{(\bullet)} \in \mathcal{C}(t,\mathcal{S})$,*

$$\|\boldsymbol{\delta}^{(\bullet)}\|_2^2 \le \phi_0^{-1}(t,\mathcal{S},\boldsymbol{\Sigma}) \cdot \|\boldsymbol{\delta}^{(\bullet)}\|_{\boldsymbol{\Sigma}}^2.$$

Here $\phi_0(t,\mathcal{S},\boldsymbol{\Sigma}) > 0$ is a parameter depending on t, $\boldsymbol{\Sigma}$ and $\mathcal{S}$, and $\|\boldsymbol{\delta}^{(\bullet)}\|_{\boldsymbol{\Sigma}} = (\boldsymbol{\delta}^{(\bullet)\mathsf{T}}\boldsymbol{\Sigma}\boldsymbol{\delta}^{(\bullet)})^{\frac{1}{2}}$.

[1]Yin Xia is the corresponding author.

S1.1 Proof of Lemma 1

Proof. First, by Assumption 4(a) or 4(b), there exists positive constants c_4 and C_4 such that with probability at least $1 - c_4 M/p$,

$$\max_{i,j,m} |X_{ij}^{(\mathsf{m})}| \leq C_4(\log pN)^{a_0}, \qquad \text{where } a_0 = 1/2 \text{ under 4(a) and } a_0 = 0 \text{ under 4(b).}$$

Let $\widehat{\mathcal{L}}^{(\mathsf{m})}_{\text{-}k,k'}(\boldsymbol{\beta}^{(\mathsf{m})}) = \widehat{\mathscr{P}}_{\mathcal{I}^{(\mathsf{m})}_{\text{-}k,k'}} f(\mathbf{X}^\mathsf{T}\boldsymbol{\beta}^{(\mathsf{m})}, Y)$ and we expand $\nabla\widehat{\mathcal{L}}^{(\mathsf{m})}_{\text{-}k,k'}(\widehat{\boldsymbol{\beta}}^{(\mathsf{m})}_{[\text{-}k,\text{-}k']})$ around $\boldsymbol{\beta}_0^{(\mathsf{m})}$ to obtain

$$\begin{aligned}\nabla\widehat{\mathcal{L}}^{(\mathsf{m})}_{\text{-}k,k'}(\widehat{\boldsymbol{\beta}}^{(\mathsf{m})}_{[\text{-}k,\text{-}k']}) &= \nabla\widehat{\mathcal{L}}^{(\mathsf{m})}_{\text{-}k,k'}(\boldsymbol{\beta}_0^{(\mathsf{m})}) + \int_0^1 \nabla^2\widehat{\mathcal{L}}^{(\mathsf{m})}_{\text{-}k,k'}\left(\boldsymbol{\beta}_0^{(\mathsf{m})} + t[\widehat{\boldsymbol{\beta}}^{(\mathsf{m})}_{[\text{-}k,\text{-}k']} - \boldsymbol{\beta}_0^{(\mathsf{m})}]\right)(\widehat{\boldsymbol{\beta}}^{(\mathsf{m})}_{[\text{-}k,\text{-}k']} - \boldsymbol{\beta}_0^{(\mathsf{m})})dt \\ &= \nabla\widehat{\mathcal{L}}^{(\mathsf{m})}_{\text{-}k,k'}(\boldsymbol{\beta}_0^{(\mathsf{m})}) + \widehat{\mathbb{H}}^{(\mathsf{m})}_{[\text{-}k,k']}(\widehat{\boldsymbol{\beta}}^{(\mathsf{m})}_{[\text{-}k,\text{-}k']} - \boldsymbol{\beta}_0^{(\mathsf{m})}) + \boldsymbol{v}^{(\mathsf{m})}_{k,k'},\end{aligned}$$

where $\widehat{\mathbb{H}}^{(\mathsf{m})}_{[\text{-}k,k']} = \widehat{\mathscr{P}}_{\mathcal{I}^{(\mathsf{m})}_{\text{-}k,k'}} \mathbf{X}_{\widehat{\boldsymbol{\beta}}^{(\mathsf{m})}_{[\text{-}k,\text{-}k']}} \mathbf{X}^\mathsf{T}_{\widehat{\boldsymbol{\beta}}^{(\mathsf{m})}_{[\text{-}k,\text{-}k']}}$, and

$$\boldsymbol{v}^{(\mathsf{m})}_{k,k'} = \int_0^1 \left\{\nabla^2\widehat{\mathcal{L}}^{(\mathsf{m})}_{\text{-}k,k'}\left(\boldsymbol{\beta}_0^{(\mathsf{m})} + t[\widehat{\boldsymbol{\beta}}^{(\mathsf{m})}_{[\text{-}k,\text{-}k']} - \boldsymbol{\beta}_0^{(\mathsf{m})}]\right) - \widehat{\mathbb{H}}^{(\mathsf{m})}_{[\text{-}k,k']}\right\}(\widehat{\boldsymbol{\beta}}^{(\mathsf{m})}_{[\text{-}k,\text{-}k']} - \boldsymbol{\beta}_0^{(\mathsf{m})})dt.$$

To bound $\boldsymbol{v}^{(\mathsf{m})}_{k,k'}$, we note that under Assumptions 2 and 4(a) or 4(b), there exists constants $c_4, C_4 > 0$ such that with probability at least $1 - c_4 M/p$,

$$\begin{aligned}&\left\|\int_0^1 \left\{\nabla^2\widehat{\mathcal{L}}^{(\mathsf{m})}_{\text{-}k,k'}\left(\boldsymbol{\beta}_0^{(\mathsf{m})} + t[\widehat{\boldsymbol{\beta}}^{(\mathsf{m})}_{[\text{-}k,\text{-}k']} - \boldsymbol{\beta}_0^{(\mathsf{m})}]\right) - \widehat{\mathbb{H}}^{(\mathsf{m})}_{[\text{-}k,k']}\right\}(\widehat{\boldsymbol{\beta}}^{(\mathsf{m})}_{[\text{-}k,\text{-}k']} - \boldsymbol{\beta}_0^{(\mathsf{m})})dt\right\|_\infty \\ &\leq \max_{t\in[0,1]}\left\|\left\{\nabla^2\widehat{\mathcal{L}}^{(\mathsf{m})}_{\text{-}k,k'}\left(\boldsymbol{\beta}_0^{(\mathsf{m})} + t[\widehat{\boldsymbol{\beta}}^{(\mathsf{m})}_{[\text{-}k,\text{-}k']} - \boldsymbol{\beta}_0^{(\mathsf{m})}]\right) - \widehat{\mathbb{H}}^{(\mathsf{m})}_{[\text{-}k,k']}\right\}(\widehat{\boldsymbol{\beta}}^{(\mathsf{m})}_{[\text{-}k,\text{-}k']} - \boldsymbol{\beta}_0^{(\mathsf{m})})\right\|_\infty \\ &\leq \max_{i,j,m}|X_{ij}^{(\mathsf{m})}| \cdot \max_{t\in[0,1]} \widehat{\mathscr{P}}_{\mathcal{I}^{(\mathsf{m})}_{\text{-}k,k'}}\left\{\left|\mathbf{X}^\mathsf{T}(\widehat{\boldsymbol{\beta}}^{(\mathsf{m})}_{[\text{-}k,\text{-}k']} - \boldsymbol{\beta}_0^{(\mathsf{m})})\right| \cdot C_L\left|(1-t)\mathbf{X}^\mathsf{T}(\widehat{\boldsymbol{\beta}}^{(\mathsf{m})}_{[\text{-}k,\text{-}k']} - \boldsymbol{\beta}_0^{(\mathsf{m})})\right|\right\} \\ &\leq C_4(\log pN)^{a_0} \cdot \widehat{\mathscr{P}}_{\mathcal{I}^{(\mathsf{m})}_{\text{-}k,k'}}\left\{\left\|\mathbf{X}^\mathsf{T}(\widehat{\boldsymbol{\beta}}^{(\mathsf{m})}_{[\text{-}k,\text{-}k']} - \boldsymbol{\beta}_0^{(\mathsf{m})})\right\|_2^2\right\}.\end{aligned}$$

Then we note that when $\widehat{\boldsymbol{\beta}}^{(\mathsf{m})}_{[\text{-}k,\text{-}k']}$ is independent of $\mathbf{X}_i^{(\mathsf{m})}$ for $i \in \mathcal{I}^{(\mathsf{m})}_{\text{-}k,k'}$, $\mathbf{X}_i^{(\mathsf{m})\mathsf{T}}(\widehat{\boldsymbol{\beta}}^{(\mathsf{m})}_{[\text{-}k,\text{-}k']} - \boldsymbol{\beta}_0^{(\mathsf{m})})$ is sub-gaussian and $\mathsf{E}\left\|\mathbf{X}_i^{(\mathsf{m})\mathsf{T}}(\widehat{\boldsymbol{\beta}}^{(\mathsf{m})}_{[\text{-}k,\text{-}k']} - \boldsymbol{\beta}_0^{(\mathsf{m})})\right\|_2^2 \leq C_3 C_\Lambda s \log p/n_m$ for all $m \in [M]$ with probability

$1 - c_3 M/p$ by Lemma S1. Thus there exists $c_5, C_5 > 0$ such that

$$\left\|\boldsymbol{v}^{(\mathsf{m})}_{k,k'}\right\|_\infty \le \frac{C_5 s M (\log pN)^{a_0} \log p}{N} \quad \text{with probability at least } 1 - c_5 M/p. \tag{S1}$$

Based on (3), we have

$$\begin{aligned} &|\mathcal{I}_{\text{-}k}|^{-1} \sum_{m=1}^{M} |\mathcal{I}^{(\mathsf{m})}_{\text{-}k}| (\widetilde{\boldsymbol{\beta}}^{(\mathsf{m})}_{[\text{-}k]} - \boldsymbol{\beta}^{(\mathsf{m})}_0)^{\mathsf{T}} \widehat{\mathbb{H}}^{(\mathsf{m})}_{[\text{-}k]} (\widetilde{\boldsymbol{\beta}}^{(\mathsf{m})}_{[\text{-}k]} - \boldsymbol{\beta}^{(\mathsf{m})}_0) + \lambda_N \left\|\widetilde{\boldsymbol{\beta}}^{(\bullet)}_{[\text{-}k],\text{-}1}\right\|_{2,1} \\ \le& -2|\mathcal{I}_{\text{-}k}|^{-1} \sum_{m=1}^{M} |\mathcal{I}^{(\mathsf{m})}_{\text{-}k}| (\widetilde{\boldsymbol{\beta}}^{(\mathsf{m})}_{[\text{-}k]} - \boldsymbol{\beta}^{(\mathsf{m})}_0)^{\mathsf{T}} (K')^{-1} \sum_{k'=1}^{K'} \left[\nabla \widehat{\mathcal{L}}^{(\mathsf{m})}_{\text{-}k,k'}(\boldsymbol{\beta}^{(\mathsf{m})}_0) + \boldsymbol{v}^{(\mathsf{m})}_{k,k'}\right] + \lambda_N \|\boldsymbol{\beta}^{(\bullet)}_0\|_{2,1}. \end{aligned} \tag{S2}$$

We next follow procedures similar to Huang and Zhang (2010); Lounici et al. (2011); Negahban et al. (2012) to derive the bound for $\widetilde{\boldsymbol{\beta}}^{(\bullet)}_{[\text{-}k]} - \boldsymbol{\beta}^{(\bullet)}_0$. First, by Lemma S1 and the sparsity condition, $\|\widehat{\boldsymbol{\beta}}^{(\mathsf{m})}_{[\text{-}k,\text{-}k']} - \boldsymbol{\beta}^{(\mathsf{m})}_0\|_2$ is bounded by any absolute constant when N is sufficiently large. From Lemma S2 and the fact $K' = \mathbb{O}(1)$, there exists a constant ϕ_0, such that $\widehat{\mathbb{H}}^{(\bullet)}_{[\text{-}k]}$ satisfies $\mathscr{C}_{\mathsf{RE}}$ on any $|\mathcal{S}| \le s$ with parameter $\phi_0\{t, \mathcal{S}, \widehat{\mathbb{H}}^{(\bullet)}_{[\text{-}k]}\} \ge \phi_0$ when N is sufficiently large. By Assumption 3, there exists constant $c_6, C_6 > 0$ that

$$\frac{1}{\sqrt{M}} \left\|\nabla \widehat{\mathcal{L}}^{(\bullet)}_{k,k'}(\boldsymbol{\beta}^{(\bullet)}_0)\right\|_{2,\infty} \le C_6 \sqrt{\frac{1 + M^{-1}\log p}{n}} \quad \text{with probability at least } 1 - c_6/p,$$

where $\nabla \widehat{\mathcal{L}}^{(\bullet)}_{k,k'}(\boldsymbol{\beta}^{(\bullet)}_0) = \{\widehat{\mathcal{L}}^{(1)\mathsf{T}}_{k,k'}(\boldsymbol{\beta}^{(1)}_0), \ldots, \widehat{\mathcal{L}}^{(\mathsf{m})\mathsf{T}}_{\text{-}k,k'}(\boldsymbol{\beta}^{(\mathsf{M})}_0)\}^{\mathsf{T}}$. Combining this with (S1), we have

$$\left\|\nabla \widehat{\mathcal{L}}^{(\bullet)}_{k,k'}(\boldsymbol{\beta}^{(\bullet)}_0) + \boldsymbol{v}^{(\bullet)}_{k,k'}\right\|_{2,\infty} \le C_6 \sqrt{\frac{M + \log p}{n}} + \frac{C_5 s M^{\frac{1}{2}} (\log pN)^{a_0} \log p}{n}.$$

Then we take $\lambda = 2M^{-1} \|\nabla \widehat{\mathcal{L}}^{(\bullet)}_{k,k'}(\boldsymbol{\beta}^{(\bullet)}_0) + \boldsymbol{v}^{(\bullet)}_{k,k'}\|_{2,\infty}$, which has the same rate as that given in Lemma 1. Adopting similar techniques used in Lounici et al. (2011); Negahban et al. (2012); Cai et al. (2019), we can prove that with probability converging to 1,

$$\|\widetilde{\boldsymbol{\beta}}^{(\bullet)}_{[\text{-}k]} - \boldsymbol{\beta}^{(\bullet)}_0\|_{2,1} \le C_8 s M \lambda_N \quad \text{and} \quad \|\widetilde{\boldsymbol{\beta}}^{(\bullet)}_{[\text{-}k]} - \boldsymbol{\beta}^{(\bullet)}_0\|_2^2 \le C_8 s M^2 \lambda_N^2, \quad \text{for some constant } C_8 > 0.$$

$\square$

S1.2 Proof of Lemma 2

Proof. From linearized expression of $Y_i^{(\mathsf{m})}$ given in section 2.3, we may write $\breve{\beta}_j^{(\mathsf{m})} - \beta_{0,j}^{(\mathsf{m})} = V_j^{(\mathsf{m})} + \Delta_{j1}^{(\mathsf{m})} + \Delta_{j2}^{(\mathsf{m})} + \Delta_{j3}^{(\mathsf{m})}$ with

$$
\begin{aligned}
V_j^{(\mathsf{m})} =& K^{-1}\sum_{k=1}^{K} \widehat{\mathscr{P}}_{\mathcal{I}_k^{(\mathsf{m})}} \boldsymbol{u}_{0,j}^{(\mathsf{m})\mathsf{T}}\mathbf{X}\epsilon, \quad \Delta_{j1}^{(\mathsf{m})} = K^{-1}\sum_{k=1}^{K} \widehat{\mathscr{P}}_{\mathcal{I}_k^{(\mathsf{m})}} \left(\widehat{\boldsymbol{u}}_{j,[k]}^{(\mathsf{m})} - \boldsymbol{u}_{0,j}^{(\mathsf{m})}\right)^{\mathsf{T}} \mathbf{X}\epsilon \\
\Delta_{j2}^{(\mathsf{m})} =& K^{-1}\sum_{k=1}^{K} \left\{\widehat{\boldsymbol{u}}_{j,[k]}^{(\mathsf{m})}\widetilde{\mathbb{H}}_{[k]}^{(\mathsf{m})} - \boldsymbol{e}_j\right\}(\boldsymbol{\beta}_0^{(\mathsf{m})} - \widetilde{\boldsymbol{\beta}}_{[\text{-}k]}^{(\mathsf{m})}), \quad \Delta_{j3}^{(\mathsf{m})} = K^{-1}\sum_{k=1}^{K} \widehat{\mathscr{P}}_{\mathcal{I}_k^{(\mathsf{m})}} \left\{\widehat{\boldsymbol{u}}_{j,[k]}^{(\mathsf{m})\mathsf{T}}\mathbf{X}R(\mathbf{X}^{\mathsf{T}}\widetilde{\boldsymbol{\beta}}_{[\text{-}k]}^{(\mathsf{m})})\right\},
\end{aligned}
$$

where $R(\cdot)$ is the remainder term defined in Section 2.1. We next bound $\sum_{m=1}^{M}|\Delta_{jt}^{(\mathsf{m})}|$ for $t = 1, 2, 3$ separately. First, for $|\Delta_{j2}^{(\mathsf{m})}|$ and $|\Delta_{j3}^{(\mathsf{m})}|$, by Lemma 1 and (4) in the paper, we have

$$
\begin{aligned}
\sum_{m=1}^{M}|\Delta_{j2}^{(\mathsf{m})}| \leq& K^{-1}\sum_{k=1}^{K}\left\|\widehat{\boldsymbol{u}}_{j,[k]}^{(\bullet)}\widetilde{\mathbb{H}}_{[k]}^{(\bullet)} - \boldsymbol{e}_j\right\|_{2,\infty}\left\|\boldsymbol{\beta}_0^{(\bullet)} - \widetilde{\boldsymbol{\beta}}_{[\text{-}k]}^{(\bullet)}\right\|_{2,1} \\
=& O_{\mathsf{P}}\left\{\left(\frac{M+\log p}{n}\right)^{\frac{1}{2}}\right\}\cdot O_{\mathsf{P}}\left\{s\left(\frac{M+\log p}{n}\right)^{\frac{1}{2}} + \frac{s^2M^{\frac{1}{2}}(\log pN)^{a_0}\log p}{n}\right\} \qquad \text{(S3)} \\
=& O_{\mathsf{P}}\left\{\frac{s(M+\log p)}{n} + \frac{s^2M^{\frac{1}{2}}(M+\log p)^{\frac{1}{2}}(\log pN)^{a_0}\log p}{n^{\frac{3}{2}}}\right\},
\end{aligned}
$$

uniformly for all $j = 2, \dots, p$ and that

$$
\sum_{m=1}^{M}|\Delta_{j3}^{(\mathsf{m})}| \leq K^{-1}\max_{i,j,m}|X_{ij}^{(\mathsf{m})}|\max_{k,m}\|\widehat{\boldsymbol{u}}_{j,[k]}^{(\mathsf{m})}\|_1\sum_{k=1}^{K}\sum_{m=1}^{M}\widehat{\mathscr{P}}_{\mathcal{I}_k^{(\mathsf{m})}}R(\mathbf{X}^{\mathsf{T}}\widetilde{\boldsymbol{\beta}}_{[\text{-}k]}^{(\mathsf{m})}),
$$

respectively. By Assumption 2 and mean value theorem, for $i \in \mathcal{I}_k^{(\mathsf{m})}$, there exists $\breve{\theta}_{ki}^{(\mathsf{m})}$ lying between $\mathbf{X}_i^{(\mathsf{m})\mathsf{T}}\boldsymbol{\beta}_0^{(\mathsf{m})}$ and $\mathbf{X}_i^{(\mathsf{m})\mathsf{T}}\widetilde{\boldsymbol{\beta}}_{[\text{-}k]}^{(\mathsf{m})}$, such that

$$
\begin{aligned}
|R_i^{(\mathsf{m})}(\mathbf{X}_i^{(\mathsf{m})\mathsf{T}}\widetilde{\boldsymbol{\beta}}_{[\text{-}k]}^{(\mathsf{m})})| =& \left|\dot{\phi}(\mathbf{X}_i^{(\mathsf{m})\mathsf{T}}\boldsymbol{\beta}_0^{(\mathsf{m})}) - \dot{\phi}(\mathbf{X}_i^{(\mathsf{m})\mathsf{T}}\widetilde{\boldsymbol{\beta}}_{[\text{-}k]}^{(\mathsf{m})}) - \ddot{\phi}(\mathbf{X}_i^{(\mathsf{m})\mathsf{T}}\widetilde{\boldsymbol{\beta}}_{[\text{-}k]}^{(\mathsf{m})})\mathbf{X}_i^{(\mathsf{m})\mathsf{T}}\left(\boldsymbol{\beta}_0^{(\mathsf{m})} - \widetilde{\boldsymbol{\beta}}_{[\text{-}k]}^{(\mathsf{m})}\right)\right| \\
=& \left|\ddot{\phi}(\mathbf{X}_i^{(\mathsf{m})\mathsf{T}}\widetilde{\boldsymbol{\beta}}_{[\text{-}k]}^{(\mathsf{m})}) - \ddot{\phi}(\breve{\theta}_{ki}^{(\mathsf{m})})\right|\left|\mathbf{X}_i^{(\mathsf{m})\mathsf{T}}\left(\boldsymbol{\beta}_0^{(\mathsf{m})} - \widetilde{\boldsymbol{\beta}}_{[\text{-}k]}^{(\mathsf{m})}\right)\right| \leq C_L\left\{\mathbf{X}_i^{(\mathsf{m})\mathsf{T}}\left(\boldsymbol{\beta}_0^{(\mathsf{m})} - \widetilde{\boldsymbol{\beta}}_{[\text{-}k]}^{(\mathsf{m})}\right)\right\}^2.
\end{aligned}
$$

Since $\mathbf{X}_i^{(m)}$ is sub-gaussian and $\widetilde{\boldsymbol{\beta}}_{[-k]}^{(m)}$ is independent of $\{\mathbf{X}_i^{(m)}, i \in \mathcal{I}_k^{(m)}\}$, it follows from concentration bounds like Theorem 3.4 in Kuchibhotla and Chakrabortty (2018) that

$$\begin{aligned}
&\sum_{k=1}^{K}\sum_{m=1}^{M} \widehat{\mathscr{P}}_{\mathcal{I}_k^{(m)}} R(\mathbf{X}^{\intercal}\widetilde{\boldsymbol{\beta}}_{[-k]}^{(m)}) \leq C_L \sum_{k=1}^{K}\sum_{m=1}^{M} \widehat{\mathscr{P}}_{\mathcal{I}_k^{(m)}} \left\{\mathbf{X}^{\intercal}\left(\boldsymbol{\beta}_0^{(m)} - \widetilde{\boldsymbol{\beta}}_{[-k]}^{(m)}\right)\right\}^2 \\
\leq & C_L \sum_{k=1}^{K}\sum_{m=1}^{M} \mathsf{E}\left[\left\{\mathbf{X}_i^{(m)\intercal}\left(\boldsymbol{\beta}_0^{(m)} - \widetilde{\boldsymbol{\beta}}_{[-k]}^{(m)}\right)\right\}^2 \middle| \widetilde{\boldsymbol{\beta}}_{[-k]}^{(m)}\right]\left(1 + O_{\mathsf{P}}\{n^{-\frac{1}{2}}\}\right) \\
= & \left(C_L + O_{\mathsf{P}}\{n^{-\frac{1}{2}}\}\right)\sum_{k=1}^{K}\sum_{m=1}^{M} (\boldsymbol{\beta}_0^{(m)} - \widetilde{\boldsymbol{\beta}}_{[-k]}^{(m)})^{\intercal}\mathscr{P}_m(\mathbf{X}\mathbf{X}^{\intercal})(\boldsymbol{\beta}_0^{(m)} - \widetilde{\boldsymbol{\beta}}_{[-k]}^{(m)}),
\end{aligned}$$

for n is sufficiently large. It then follows that under Assumption 4(a) or 4(b), Lemma 1 and Lemma S3,

$$\begin{aligned}
\sum_{m=1}^{M} |\Delta_{j3}^{(m)}| = & O_{\mathsf{P}}\{(\log pN)^{a_0}\} \cdot O_{\mathsf{P}}\left(\left\|\boldsymbol{\beta}_0^{(\bullet)} - \widetilde{\boldsymbol{\beta}}_{[-k]}^{(\bullet)}\right\|_2^2\right) \\
= & O_{\mathsf{P}}\left\{\frac{s(\log pN)^{a_0}(M + \log p)}{n} + \frac{s^3 M(\log p)^2(\log pN)^{3a_0}}{n^2}\right\},
\end{aligned} \tag{S4}$$

uniformly for all $j = 2, \ldots, p$. We next derive the rate of $\sum_{m=1}^{M} |\Delta_{j1}^{(m)}|$. Since $\widehat{\boldsymbol{u}}_{j,[k]}^{(m)}$ only depends on $\{\mathbf{X}_i^{(m)}, i \in \mathcal{I}_k^{(m)}\}$ and data complement to the fold k, we have $\mathsf{E}(\epsilon_i^{(m)} | \widehat{\boldsymbol{u}}_{j,[k]}^{(m)}, \mathbf{X}_i^{(m)}) = 0$ when $i \in \mathcal{I}_k^{(m)}$. Thus

$$\mathsf{E}\left\{\widehat{\mathscr{P}}_{\mathcal{I}_k^{(m)}}(\widehat{\boldsymbol{u}}_{j,[k]}^{(m)} - \boldsymbol{u}_{0,j}^{(m)})^{\intercal}\mathbf{X}\epsilon \middle| \mathbf{X}, \widehat{\boldsymbol{u}}_{j,[k]}^{(m)}\right\} = 0. \tag{S5}$$

We denote the conditional variance of $(n/K)^{\frac{1}{2}}\widehat{\mathscr{P}}_{\mathcal{I}_k^{(m)}}(\widehat{\boldsymbol{u}}_{j,[k]}^{(m)} - \boldsymbol{u}_{0,j}^{(m)})^{\intercal}\mathbf{X}\epsilon$ given $\mathbf{X}^{(m)}$ and $\widehat{\boldsymbol{u}}_{j,[k]}^{(m)}$ as $\delta_{j,k}^{(m)}$ and by Assumption 3, $\delta_{j,k}^{(m)}$ satisfies

$$\delta_{j,k}^{(m)} \leq \left(\widehat{\boldsymbol{u}}_{j,[k]}^{(m)} - \boldsymbol{u}_{0,j}^{(m)}\right)^{\intercal}\left\{\widehat{\mathscr{P}}_{\mathcal{I}_k^{(m)}}\mathbf{X}\mathbf{X}^{\intercal}\ddot{\phi}(\mathbf{X}^{\intercal}\boldsymbol{\beta}_0^{(m)})\right\}\left(\widehat{\boldsymbol{u}}_{j,[k]}^{(m)} - \boldsymbol{u}_{0,j}^{(m)}\right) \cdot \max_{i,m} \ddot{\phi}^{-1}(\mathbf{X}_i^{(m)}\boldsymbol{\beta}_0^{(m)})\kappa^2(\mathbf{X}_i^{(m)}).$$

It then follows from Assumption 3 that there exists constant C_{10}, with probability 1,

$$\begin{aligned}\delta_{j,k}^{(\mathsf{m})} \leq & C_\epsilon \left(\left\| \widehat{\boldsymbol{u}}_{j,[k]}^{(\mathsf{m})} \right\|_1 + \left\| \boldsymbol{u}_{0,j}^{(\mathsf{m})} \right\|_1 \right) \cdot \left\| \left\{ \widehat{\mathscr{P}}_{\mathcal{I}_k^{(\mathsf{m})}} \mathbf{X}\mathbf{X}^\intercal \ddot{\phi}(\mathbf{X}^\intercal \boldsymbol{\beta}_0^{(\mathsf{m})}) \right\} \left(\widehat{\boldsymbol{u}}_{j,[k]}^{(\mathsf{m})} - \boldsymbol{u}_{0,j}^{(\mathsf{m})} \right) \right\|_\infty \\ \leq & C_{10} \left\| \left\{ \widehat{\mathscr{P}}_{\mathcal{I}_k^{(\mathsf{m})}} \mathbf{X}\mathbf{X}^\intercal \ddot{\phi}(\mathbf{X}^\intercal \boldsymbol{\beta}_0^{(\mathsf{m})}) \right\} \boldsymbol{u}_{0,j}^{(\mathsf{m})} - \left\{ \widehat{\mathscr{P}}_{\mathcal{I}_k^{(\mathsf{m})}} \mathbf{X}\mathbf{X}^\intercal \ddot{\phi}(\mathbf{X}^\intercal \widetilde{\boldsymbol{\beta}}_{[\text{-}k]}^{(\mathsf{m})}) \right\} \widehat{\boldsymbol{u}}_{j,[k]}^{(\mathsf{m})} \right\|_\infty + \\ & + C_{10} \left\| \widehat{\mathscr{P}}_{\mathcal{I}_k^{(\mathsf{m})}} \mathbf{X}\mathbf{X}^\intercal \{ \ddot{\phi}(\mathbf{X}^\intercal \boldsymbol{\beta}_0^{(\mathsf{m})}) - \ddot{\phi}(\mathbf{X}^\intercal \widetilde{\boldsymbol{\beta}}_{[\text{-}k]}^{(\mathsf{m})}) \} \right\|_{\max} \left\| \widehat{\boldsymbol{u}}_{j,[k]}^{(\mathsf{m})} \right\|_1 .\end{aligned} \tag{S6}$$

Again using Assumption 2, we have

$$\begin{aligned}& \left\| \widehat{\mathscr{P}}_{\mathcal{I}_k^{(\mathsf{m})}} \mathbf{X}\mathbf{X}^\intercal \{ \ddot{\phi}(\mathbf{X}^\intercal \boldsymbol{\beta}_0^{(\mathsf{m})}) - \ddot{\phi}(\mathbf{X}^\intercal \widetilde{\boldsymbol{\beta}}_{[\text{-}k]}^{(\mathsf{m})}) \} \right\|_{\max} \\ \leq & \max_{r,j \in [p]} \left\{ \widehat{\mathscr{P}}_{\mathcal{I}_k^{(\mathsf{m})}} |X_r X_j| \left| \ddot{\phi}(\mathbf{X}^\intercal \boldsymbol{\beta}_0^{(\mathsf{m})}) - \ddot{\phi}(\mathbf{X}^\intercal \widetilde{\boldsymbol{\beta}}_{[\text{-}k]}^{(\mathsf{m})}) \right| \right\} \leq \max_{r,j \in [p]} \left\{ \widehat{\mathscr{P}}_{\mathcal{I}_k^{(\mathsf{m})}} |X_r X_j| C_L \left| \mathbf{X}^\intercal \left(\boldsymbol{\beta}_0^{(\mathsf{m})} - \widetilde{\boldsymbol{\beta}}_{[\text{-}k]}^{(\mathsf{m})} \right) \right| \right\} \\ \leq & C_L \max_{r,j \in [p]} \left[\widehat{\mathscr{P}}_{\mathcal{I}_k^{(\mathsf{m})}} X_r^2 X_j^2 \widehat{\mathscr{P}}_{\mathcal{I}_k^{(\mathsf{m})}} \left\{ \mathbf{X}^\intercal \left(\boldsymbol{\beta}_0^{(\mathsf{m})} - \widetilde{\boldsymbol{\beta}}_{[\text{-}k]}^{(\mathsf{m})} \right) \right\}^2 \right]^{\frac{1}{2}} .\end{aligned}$$

Again using Theorem 3.4 in (Kuchibhotla and Chakrabortty, 2018) and when $n > \log p$,

$$\begin{aligned}& \left\| \widehat{\mathscr{P}}_{\mathcal{I}_k^{(\mathsf{m})}} \mathbf{X}\mathbf{X}^\intercal \{ \ddot{\phi}(\mathbf{X}^\intercal \boldsymbol{\beta}_0^{(\mathsf{m})}) - \ddot{\phi}(\mathbf{X}^\intercal \widetilde{\boldsymbol{\beta}}_{[\text{-}k]}^{(\mathsf{m})}) \} \right\|_{\max} \\ \leq & C_L \max_{r,j \in [p]} \left[\left(1 + O_{\mathsf{P}} \left\{ \frac{(\log p)^{\frac{1}{2}}}{n^{\frac{1}{2}}} \right\} \right) \{ \mathscr{P}^{(\mathsf{m})} |X_r^2 X_j^2| \} (\boldsymbol{\beta}_0^{(\mathsf{m})} - \widetilde{\boldsymbol{\beta}}_{[\text{-}k]}^{(\mathsf{m})})^\intercal \left\{ \mathscr{P}^{(\mathsf{m})} (\mathbf{X}\mathbf{X}^\intercal) \right\} (\boldsymbol{\beta}_0^{(\mathsf{m})} - \widetilde{\boldsymbol{\beta}}_{[\text{-}k]}^{(\mathsf{m})}) \right]^{\frac{1}{2}} \\ = & O_{\mathsf{P}} \left\{ \frac{s^{\frac{1}{2}} (M + \log p)^{\frac{1}{2}}}{n^{\frac{1}{2}}} + \frac{s^{\frac{3}{2}} M^{\frac{1}{2}} (\log pN)^{a_0} \log p}{n} \right\} .\end{aligned} \tag{S7}$$

It can be verified that

$$\frac{s^3 M (\log pN)^{2a_0} (\log p)^2}{n^2} \leq O \left\{ \frac{s(M + \log p)}{n} \right\}, \text{ as } s = o \left\{ \frac{n^{\frac{1}{2}}}{(M + \log p)(\log pN)^{a_0} (\log p)^{\frac{1}{2}}} \right\} .$$

By the proof of Lemma S3, it then follows that

$$\left\|\left\{\widehat{\mathscr{P}}_{\mathcal{I}_k^{(\mathrm{m})}}\mathbf{X}\mathbf{X}^{\intercal}\ddot{\phi}(\mathbf{X}^{\intercal}\boldsymbol{\beta}_0^{(\mathrm{m})})\right\}\boldsymbol{u}_{0,j}^{(\mathrm{m})}-\left\{\widehat{\mathscr{P}}_{\mathcal{I}_k^{(\mathrm{m})}}\mathbf{X}\mathbf{X}^{\intercal}\ddot{\phi}(\mathbf{X}^{\intercal}\widetilde{\boldsymbol{\beta}}_{[\text{-}k]}^{(\mathrm{m})})\right\}\widehat{\boldsymbol{u}}_{j,[k]}^{(\mathrm{m})}\right\|_{\infty}=O_{\mathsf{P}}\left\{\frac{s^{\frac{1}{2}}(M+\log p)^{\frac{1}{2}}}{n^{\frac{1}{2}}}\right\}.$$

Consequently $\delta_{j,k}^{(\mathrm{m})}=O_{\mathsf{P}}\left\{s^{\frac{1}{2}}(M+\log p)^{\frac{1}{2}}n^{-\frac{1}{2}}\right\}$ by Lemma S3. Combining this with (S5) and the concentration bound, we have that uniformly for all $j=2,\dots,p$,

$$\sum_{m=1}^{M}|\Delta_{j1}^{(\mathrm{m})}|=M\cdot O_{\mathsf{P}}\left\{\frac{s^{\frac{1}{4}}(M+\log p)^{\frac{1}{4}}}{n^{\frac{1}{4}}}\cdot\frac{(\log p)^{\frac{1}{2}}}{n^{\frac{1}{2}}}\right\}=O_{\mathsf{P}}\left\{\frac{s^{\frac{1}{4}}M(\log p)^{\frac{1}{2}}(M+\log p)^{\frac{1}{4}}}{n^{\frac{3}{4}}}\right\}.$$

Combining this with (S3), (S4) and the assumption that

$$s=o\left\{\frac{n^{\frac{1}{2}}}{(\log pN)^{a_0}(M+\log p)(\log p)^{\frac{1}{2}}}\wedge\frac{n}{M^4(\log p)^4(M+\log p)}\right\},$$

we can derive the rate for the bias term $\sum_{m=1}^{M}|\Delta_j^{(\mathrm{m})}|$:

$$\begin{aligned}\sum_{m=1}^{M}|\Delta_j^{(\mathrm{m})}|\le&\sum_{m=1}^{M}(|\Delta_{j1}^{(\mathrm{m})}|+|\Delta_{j2}^{(\mathrm{m})}|+|\Delta_{j3}^{(\mathrm{m})}|)\\ =&O_{\mathsf{P}}\left\{\frac{s^{\frac{1}{4}}M(\log p)^{\frac{1}{2}}(M+\log p)^{\frac{1}{4}}}{n^{\frac{3}{4}}}\right\}+O_{\mathsf{P}}\left\{\frac{s^2M^{\frac{1}{2}}(\log pN)^{a_0}(M+\log p)^{\frac{1}{2}}\log p}{n^{\frac{3}{2}}}\right\}\\ &+O_{\mathsf{P}}\left\{\frac{s(\log pN)^{a_0}(M+\log p)}{n}+\frac{s^3M(\log pN)^{3a_0}(\log p)^2}{n^2}\right\}=o_{\mathsf{P}}\left\{\frac{1}{(n\log p)^{\frac{1}{2}}}\right\},\end{aligned}$$

In above equation, we again use that as $s=o\left\{n^{\frac{1}{2}}(\log p)^{-\frac{1}{2}}(\log pN)^{-a_0}(M+\log p)^{-1}\right\}$,

$$\begin{aligned}\frac{s^2M^{\frac{1}{2}}(\log pN)^{a_0}(M+\log p)^{\frac{1}{2}}\log p}{n^{\frac{3}{2}}}&\le O\left\{\frac{s(\log pN)^{a_0}(M+\log p)}{n}\right\};\\ \text{and}\quad\frac{s^3M(\log pN)^{3a_0}(\log p)^2}{n^2}&\le O\left\{\frac{s(\log pN)^{a_0}(M+\log p)}{n}\right\}.\end{aligned}$$

Then we finish showing the result for $\sum_{m=1}^{M}|\Delta_j^{(\mathrm{m})}|$. At last, we prove that $\left|(\widehat{\sigma}_j^{(\mathrm{m})})^2-(\sigma_{0,j}^{(\mathrm{m})})^2\right|=o_{\mathsf{P}}\left\{(\log p)^{-1}\right\}$ uniformly for all $j=2,\dots,p$. Recalling that $(\widehat{\sigma}_j^{(\mathrm{m})})^2=K^{-1}\sum_{k=1}^{K}\widehat{\boldsymbol{u}}_{j,[k]}^{(\mathrm{m})\intercal}\widetilde{\mathbb{J}}_{[k]}^{(\mathrm{m})}\widehat{\boldsymbol{u}}_{j,[k]}^{(\mathrm{m})}$,

we only need to prove that $\left|\widehat{\boldsymbol{u}}_{j,[k]}^{(\mathsf{m})\mathsf{T}}\widetilde{\mathbb{J}}_{[k]}^{(\mathsf{m})}\widehat{\boldsymbol{u}}_{j,[k]}^{(\mathsf{m})}-(\sigma_{0,j}^{(\mathsf{m})})^2\right|=o_{\mathsf{P}}\left\{(\log p)^{-1}\right\}$. To prove this, we let $\widehat{\mathscr{E}}_{j,[k]}^{(\mathsf{m})}=\widehat{\boldsymbol{u}}_{j,[k]}^{(\mathsf{m})}\mathbf{X}\mathbf{X}^{\mathsf{T}}\widehat{\boldsymbol{u}}_{j,[k]}^{(\mathsf{m})}$ and first note that

$$
\begin{aligned}
&\left|\widehat{\boldsymbol{u}}_{j,[k]}^{(\mathsf{m})\mathsf{T}}\widetilde{\mathbb{J}}_{[-k]}^{(\mathsf{m})}\widehat{\boldsymbol{u}}_{j,[k]}^{(\mathsf{m})}-\widehat{\mathscr{P}}_{\mathcal{I}_k^{(\mathsf{m})}}\widehat{\boldsymbol{u}}_{j,[k]}^{(\mathsf{m})}\mathbf{X}\mathbf{X}^{\mathsf{T}}\widehat{\boldsymbol{u}}_{j,[k]}^{(\mathsf{m})}\left\{Y-\dot{\phi}(\mathbf{X}^{\mathsf{T}}\boldsymbol{\beta}_0^{(\mathsf{m})})\right\}^2\right| \\
\leq&2\left|\widehat{\mathscr{P}}_{\mathcal{I}_k^{(\mathsf{m})}}\widehat{\boldsymbol{u}}_{j,[k]}^{(\mathsf{m})}\mathbf{X}\mathbf{X}^{\mathsf{T}}\widehat{\boldsymbol{u}}_{j,[k]}^{(\mathsf{m})}\left\{Y-\dot{\phi}(\mathbf{X}^{\mathsf{T}}\boldsymbol{\beta}_0^{(\mathsf{m})})\right\}\left\{\dot{\phi}(\mathbf{X}^{\mathsf{T}}\boldsymbol{\beta}_0^{(\mathsf{m})})-\dot{\phi}(\mathbf{X}^{\mathsf{T}}\widetilde{\boldsymbol{\beta}}_{[-k]}^{(\mathsf{m})})\right\}\right| \\
&+\left|\widehat{\mathscr{P}}_{\mathcal{I}_k^{(\mathsf{m})}}\widehat{\mathscr{E}}_{j,[k]}^{(\mathsf{m})}\left\{\dot{\phi}(\mathbf{X}^{\mathsf{T}}\boldsymbol{\beta}_0^{(\mathsf{m})})-\dot{\phi}(\mathbf{X}^{\mathsf{T}}\widetilde{\boldsymbol{\beta}}_{[-k]}^{(\mathsf{m})})\right\}^2\right| \qquad \text{(S8)}\\
\leq&2\left[\widehat{\mathscr{P}}_{\mathcal{I}_k^{(\mathsf{m})}}\widehat{\mathscr{E}}_{j,[k]}^{(\mathsf{m})}\left\{Y-\dot{\phi}(\mathbf{X}^{\mathsf{T}}\boldsymbol{\beta}_0^{(\mathsf{m})})\right\}^2\right]^{\frac{1}{2}}\left[\widehat{\mathscr{P}}_{\mathcal{I}_k^{(\mathsf{m})}}\widehat{\mathscr{E}}_{j,[k]}^{(\mathsf{m})}\left\{\dot{\phi}(\mathbf{X}^{\mathsf{T}}\boldsymbol{\beta}_0^{(\mathsf{m})})-\dot{\phi}(\mathbf{X}^{\mathsf{T}}\widetilde{\boldsymbol{\beta}}_{[-k]}^{(\mathsf{m})})\right\}^2\right]^{\frac{1}{2}} \\
&+\left|\widehat{\mathscr{P}}_{\mathcal{I}_k^{(\mathsf{m})}}\widehat{\mathscr{E}}_{j,[k]}^{(\mathsf{m})}\left\{\dot{\phi}(\mathbf{X}^{\mathsf{T}}\boldsymbol{\beta}_0^{(\mathsf{m})})-\dot{\phi}(\mathbf{X}^{\mathsf{T}}\widetilde{\boldsymbol{\beta}}_{[-k]}^{(\mathsf{m})})\right\}^2\right|.
\end{aligned}
$$

Using Taylor series expansion, there exists $\breve{\theta}_{ki}^{(\mathsf{m})}$ lying between $\mathbf{X}_i^{(\mathsf{m})\mathsf{T}}\boldsymbol{\beta}_0^{(\mathsf{m})}$ and $\mathbf{X}_i^{(\mathsf{m})\mathsf{T}}\widetilde{\boldsymbol{\beta}}_{[-k]}^{(\mathsf{m})}$,

$$
\begin{aligned}
&\left|\dot{\phi}(\mathbf{X}_i^{(\mathsf{m})\mathsf{T}}\boldsymbol{\beta}_0^{(\mathsf{m})})-\dot{\phi}(\mathbf{X}_i^{(\mathsf{m})\mathsf{T}}\widetilde{\boldsymbol{\beta}}_{[-k]}^{(\mathsf{m})})\right|=\left|\ddot{\phi}(\breve{\theta}_{ki}^{(\mathsf{m})})\left(\mathbf{X}_i^{(\mathsf{m})\mathsf{T}}\boldsymbol{\beta}_0^{(\mathsf{m})}-\mathbf{X}_i^{(\mathsf{m})\mathsf{T}}\widetilde{\boldsymbol{\beta}}_{[-k]}^{(\mathsf{m})}\right)\right| \\
\leq&\left|\ddot{\phi}(\breve{\theta}_{ki}^{(\mathsf{m})})-\ddot{\phi}(\mathbf{X}_i^{(\mathsf{m})\mathsf{T}}\boldsymbol{\beta}_0^{(\mathsf{m})})\right|\left|\mathbf{X}_i^{(\mathsf{m})\mathsf{T}}\boldsymbol{\beta}_0^{(\mathsf{m})}-\mathbf{X}_i^{(\mathsf{m})\mathsf{T}}\widetilde{\boldsymbol{\beta}}_{[-k]}^{(\mathsf{m})}\right|+\ddot{\phi}(\mathbf{X}_i^{(\mathsf{m})\mathsf{T}}\boldsymbol{\beta}_0^{(\mathsf{m})})\left|\mathbf{X}_i^{(\mathsf{m})\mathsf{T}}\boldsymbol{\beta}_0^{(\mathsf{m})}-\mathbf{X}_i^{(\mathsf{m})\mathsf{T}}\widetilde{\boldsymbol{\beta}}_{[-k]}^{(\mathsf{m})}\right| \\
\leq&C_L\left(\mathbf{X}_i^{(\mathsf{m})\mathsf{T}}\boldsymbol{\beta}_0^{(\mathsf{m})}-\mathbf{X}_i^{(\mathsf{m})\mathsf{T}}\widetilde{\boldsymbol{\beta}}_{[-k]}^{(\mathsf{m})}\right)^2+\ddot{\phi}(\mathbf{X}_i^{(\mathsf{m})\mathsf{T}}\boldsymbol{\beta}_0^{(\mathsf{m})})\left|\mathbf{X}_i^{(\mathsf{m})\mathsf{T}}\boldsymbol{\beta}_0^{(\mathsf{m})}-\mathbf{X}_i^{(\mathsf{m})\mathsf{T}}\widetilde{\boldsymbol{\beta}}_{[-k]}^{(\mathsf{m})}\right|,
\end{aligned}
$$

where we again use Assumption 2 for the last inequality. Then similar to (S7) where we use the concentration results, using Assumptions 1, 4(a) or 4(b) and the boundness of $\left\|\widehat{\boldsymbol{u}}_{j,[k]}^{(\mathsf{m})}\right\|_1$,

we have

$$
\begin{aligned}
&\left|\widehat{\mathscr{P}}_{\mathcal{I}_k^{(\mathsf{m})}}\widehat{\boldsymbol{u}}_{j,[k]}^{(\mathsf{m})}\mathbf{X}\mathbf{X}^{\mathsf{T}}\widehat{\boldsymbol{u}}_{j,[k]}^{(\mathsf{m})}\left\{\dot{\phi}(\mathbf{X}^{\mathsf{T}}\boldsymbol{\beta}_0^{(\mathsf{m})})-\dot{\phi}(\mathbf{X}^{\mathsf{T}}\widetilde{\boldsymbol{\beta}}_{[\text{-}k]}^{(\mathsf{m})})\right\}^2\right|\\
\leq& C_L^2\left|\widehat{\mathscr{P}}_{\mathcal{I}_k^{(\mathsf{m})}}\widehat{\mathscr{E}}_{j,[k]}^{(\mathsf{m})}\left(\mathbf{X}^{\mathsf{T}}\boldsymbol{\beta}_0^{(\mathsf{m})}-\mathbf{X}^{\mathsf{T}}\widetilde{\boldsymbol{\beta}}_{[\text{-}k]}^{(\mathsf{m})}\right)^4\right|+\widehat{\mathscr{P}}_{\mathcal{I}_k^{(\mathsf{m})}}\widehat{\mathscr{E}}_{j,[k]}^{(\mathsf{m})}\ddot{\phi}^2(\mathbf{X}^{\mathsf{T}}\boldsymbol{\beta}_0^{(\mathsf{m})})\left(\mathbf{X}^{\mathsf{T}}\boldsymbol{\beta}_0^{(\mathsf{m})}-\mathbf{X}^{\mathsf{T}}\widetilde{\boldsymbol{\beta}}_{[\text{-}k]}^{(\mathsf{m})}\right)^2\\
\leq&\left\|\widehat{\boldsymbol{u}}_{j,[k]}^{(\mathsf{m})}\right\|_1^2\left(C_L^2\left\|\widehat{\mathscr{P}}_{\mathcal{I}_k^{(\mathsf{m})}}\mathbf{X}\mathbf{X}^{\mathsf{T}}\left(\mathbf{X}^{\mathsf{T}}\boldsymbol{\beta}_0^{(\mathsf{m})}-\mathbf{X}^{\mathsf{T}}\widetilde{\boldsymbol{\beta}}_{[\text{-}k]}^{(\mathsf{m})}\right)^4\right\|_{\max}+\left\|\widehat{\mathscr{P}}_{\mathcal{I}_k^{(\mathsf{m})}}\mathbf{X}\mathbf{X}^{\mathsf{T}}\ddot{\phi}^2(\mathbf{X}^{\mathsf{T}}\boldsymbol{\beta}_0^{(\mathsf{m})})\left(\mathbf{X}^{\mathsf{T}}\boldsymbol{\beta}_0^{(\mathsf{m})}-\mathbf{X}^{\mathsf{T}}\widetilde{\boldsymbol{\beta}}_{[\text{-}k]}^{(\mathsf{m})}\right)^2\right\|_{\max}\right)\\
\leq& C_L\left\|\widehat{\boldsymbol{u}}_{j,[k]}^{(\mathsf{m})}\right\|_1^2\max_{m,i}\left[\mathbf{X}_i^{(\mathsf{m})\mathsf{T}}\left(\boldsymbol{\beta}_0^{(\mathsf{m})}-\widetilde{\boldsymbol{\beta}}_{[\text{-}k]}^{(\mathsf{m})}\right)\right]^2\max_{r,j\in[p]}\left\{\mathscr{P}^{(\mathsf{m})}|X_rX_j|\right\}(\boldsymbol{\beta}_0^{(\mathsf{m})}-\widetilde{\boldsymbol{\beta}}_{[\text{-}k]}^{(\mathsf{m})})^{\mathsf{T}}\left\{\mathscr{P}^{(\mathsf{m})}\mathbf{X}\mathbf{X}^{\mathsf{T}}\right\}(\boldsymbol{\beta}_0^{(\mathsf{m})}-\widetilde{\boldsymbol{\beta}}_{[\text{-}k]}^{(\mathsf{m})})\\
&+\left\|\widehat{\boldsymbol{u}}_{j,[k]}^{(\mathsf{m})}\right\|_1^2\max_{r,j\in[p]}\left\{\mathscr{P}^{(\mathsf{m})}\left|X_rX_j\ddot{\phi}(\mathbf{X}^{\mathsf{T}}\boldsymbol{\beta}_0^{(\mathsf{m})})\right|\right\}(\boldsymbol{\beta}_0^{(\mathsf{m})}-\widetilde{\boldsymbol{\beta}}_{[\text{-}k]}^{(\mathsf{m})})^{\mathsf{T}}\left\{\mathscr{P}^{(\mathsf{m})}\mathbf{X}\mathbf{X}^{\mathsf{T}}\ddot{\phi}(\mathbf{X}^{\mathsf{T}}\boldsymbol{\beta}_0^{(\mathsf{m})})\right\}(\boldsymbol{\beta}_0^{(\mathsf{m})}-\widetilde{\boldsymbol{\beta}}_{[\text{-}k]}^{(\mathsf{m})})\\
=&O_{\mathsf{P}}\left\{1+\frac{s^2(M+\log p)\log p}{n}\right\}\cdot O_{\mathsf{P}}\left\{\frac{s(M+\log p)}{n}\right\}=O_{\mathsf{P}}\left\{\frac{s(M+\log p)}{n}\right\},
\end{aligned}
$$

using the sparsity assumption of Lemma 2 at last. Combining this with (S7), we have

$$
\begin{aligned}
&\left|\widehat{\boldsymbol{u}}_{j,[k]}^{(\mathsf{m})\mathsf{T}}\widetilde{\mathbb{J}}_{[\text{-}k]}^{(\mathsf{m})}\widehat{\boldsymbol{u}}_{j,[k]}^{(\mathsf{m})}-\widehat{\mathscr{P}}_{\mathcal{I}_k^{(\mathsf{m})}}\widehat{\mathscr{E}}_{j,[k]}^{(\mathsf{m})}\left\{Y-\dot{\phi}(\mathbf{X}^{\mathsf{T}}\boldsymbol{\beta}_0^{(\mathsf{m})})\right\}^2\right|\\
=&2O_{\mathsf{P}}\left\{\frac{s^{\frac{1}{2}}(M+\log p)^{\frac{1}{2}}}{n^{\frac{1}{2}}}\right\}\left[\widehat{\mathscr{P}}_{\mathcal{I}_k^{(\mathsf{m})}}\widehat{\mathscr{E}}_{j,[k]}^{(\mathsf{m})}\left\{Y-\dot{\phi}(\mathbf{X}^{\mathsf{T}}\boldsymbol{\beta}_0^{(\mathsf{m})})\right\}^2\right]^{\frac{1}{2}}+O_{\mathsf{P}}\left\{\frac{s(M+\log p)}{n}\right\}.
\end{aligned}
\tag{S9}
$$

Then use Assumption 3 and results in (S6) and (S7) to derive that uniformly for all m,j,k:

$$
\begin{aligned}
&\left|\widehat{\mathscr{P}}_{\mathcal{I}_k^{(\mathsf{m})}}\widehat{\boldsymbol{u}}_{j,[k]}^{(\mathsf{m})}\mathbf{X}_i^{(\mathsf{m})}\mathbf{X}_i^{(\mathsf{m})\mathsf{T}}\widehat{\boldsymbol{u}}_{j,[k]}^{(\mathsf{m})}\left\{Y_i^{(\mathsf{m})}-\dot{\phi}(\mathbf{X}_i^{(\mathsf{m})\mathsf{T}}\boldsymbol{\beta}_0^{(\mathsf{m})})\right\}^2-(\sigma_{0,j}^{(\mathsf{m})})^2\right|\\
\leq&\left|\left(\widehat{\boldsymbol{u}}_{j,[k]}^{(\mathsf{m})}-\boldsymbol{u}_{0,j}^{(\mathsf{m})}\right)^{\mathsf{T}}\left\{\widehat{\mathscr{P}}_{\mathcal{I}_k^{(\mathsf{m})}}\mathbf{X}\mathbf{X}^{\mathsf{T}}\left\{Y-\dot{\phi}(\mathbf{X}^{\mathsf{T}}\boldsymbol{\beta}_0^{(\mathsf{m})})\right\}^2\right\}\left(\widehat{\boldsymbol{u}}_{j,[k]}^{(\mathsf{m})}+\boldsymbol{u}_{0,j}^{(\mathsf{m})}\right)\right|+O_{\mathsf{P}}\left\{(n^{-1}\log p)^{1/2}\right\}\\
\leq&\left(\widehat{\boldsymbol{u}}_{j,[k]}^{(\mathsf{m})}-\boldsymbol{u}_{0,j}^{(\mathsf{m})}\right)^{\mathsf{T}}\left\{\widehat{\mathscr{P}}_{\mathcal{I}_k^{(\mathsf{m})}}\mathbf{X}\mathbf{X}^{\mathsf{T}}\ddot{\phi}(\mathbf{X}^{\mathsf{T}}\boldsymbol{\beta}_0^{(\mathsf{m})})\right\}\left(\widehat{\boldsymbol{u}}_{j,[k]}^{(\mathsf{m})}-\boldsymbol{u}_{0,j}^{(\mathsf{m})}\right)\cdot\max_{i,m}\ddot{\phi}^{-1}(\mathbf{X}_i^{(\mathsf{m})}\boldsymbol{\beta}_0^{(\mathsf{m})})\kappa^2(\mathbf{X}_i^{(\mathsf{m})})+O_{\mathsf{P}}\left\{(n^{-1}\log p)^{1/2}\right\}\\
\leq&O_{\mathsf{P}}\left(\left\|\left\{\widehat{\mathscr{P}}_{\mathcal{I}_k^{(\mathsf{m})}}\mathbf{X}\mathbf{X}^{\mathsf{T}}\ddot{\phi}(\mathbf{X}^{\mathsf{T}}\boldsymbol{\beta}_0^{(\mathsf{m})})\right\}\left(\widehat{\boldsymbol{u}}_{j,[k]}^{(\mathsf{m})}-\boldsymbol{u}_{0,j}^{(\mathsf{m})}\right)\right\|_{\infty}\right)+O_{\mathsf{P}}\left\{(n^{-1}\log p)^{1/2}\right\}=O_{\mathsf{P}}\left\{\frac{s^{\frac{1}{2}}(M+\log p)^{\frac{1}{2}}}{n^{\frac{1}{2}}}\right\},
\end{aligned}
$$

where we again use the fact that $\|\widehat{\boldsymbol{u}}_{j,[k]}^{(\mathsf{m})}\|_1$ and $\|\boldsymbol{u}_0^{(\mathsf{m})}\|_1$ are bounded by some absolute constant, as well as Theorem 3.4 in (Kuchibhotla and Chakrabortty, 2018) to concentrate the zero-mean sum as $O_{\mathsf{P}}\{(\log p)^{\frac{1}{2}}n^{-\frac{1}{2}}\}$ simultaneously. Combining this with (S9) and again using

the assumption for s, we have $\left|(\widehat{\sigma}_j^{(\mathsf{m})})^2 - (\sigma_{0,j}^{(\mathsf{m})})^2\right| = o_{\mathsf{P}}\left\{(\log p)^{-1}\right\}$. □

S1.3 Proof of Theorem 1

Proof. Let $Z_{ij}^{(\mathsf{m})} = (\boldsymbol{u}_{0,j}^{(\mathsf{m})\mathsf{T}}\mathbf{X}_i^{(\mathsf{m})})\epsilon_i^{(\mathsf{m})}/\sigma_{0,j}^{(\mathsf{m})}$ for $i \in [n_m]$,

$$W_j^{(\mathsf{m})} = n_m^{\frac{1}{2}}\frac{\breve{\beta}_j^{(\mathsf{m})}}{\widehat{\sigma}_j^{(\mathsf{m})}}, \quad \widehat{U}_j^{(\mathsf{m})} = n_m^{\frac{1}{2}}\frac{V_j^{(\mathsf{m})}}{\widehat{\sigma}_{0,j}^{(\mathsf{m})}} \quad \text{and} \quad U_j^{(\mathsf{m})} = n_m^{\frac{1}{2}}\frac{V_j^{(\mathsf{m})}}{\sigma_{0,j}^{(\mathsf{m})}} = n_m^{-\frac{1}{2}}\sum_{i=1}^{n_m} Z_{ij}^{(\mathsf{m})}.$$

To bound the difference between the test statistic $\breve{\zeta}_j = \sum_{m=1}^M (W_j^{(\mathsf{m})})^2$ and its asymptotic representation $S_j = \sum_{m=1}^M (U_j^{(\mathsf{m})})^2$, we first note that

$$\max_{m,j}|V_j^{(\mathsf{m})}| = O_{\mathsf{P}}\{(\log p)^{\frac{1}{2}}n^{-\frac{1}{2}}\}, \quad \widehat{\sigma}_j^{(\mathsf{m})} = \mathbb{O}_{\mathsf{P}}(1), \quad \sigma_{0,j}^{(\mathsf{m})} = \mathbb{O}(1).$$

Under the null $\beta_{0,j}^{(\mathsf{m})} = 0$ and using lemma 2, we have

$$\begin{aligned}
\left|\breve{\zeta}_j - S_j\right| =& \left|\sum_{m=1}^M\left\{U_j^{(\mathsf{m})} + \left(\widehat{U}_j^{(\mathsf{m})} - U_j^{(\mathsf{m})}\right) + n_m^{\frac{1}{2}}\frac{\Delta_j^{(\mathsf{m})}}{\widehat{\sigma}_j^{(\mathsf{m})}}\right\}^2 - S_j\right| \\
\leq & 2\sum_{m=1}^M\left|U_j^{(\mathsf{m})}\right|\cdot\left|\widehat{U}_j^{(\mathsf{m})} - U_j^{(\mathsf{m})}\right| + 2\sum_{m=1}^M\left|U_j^{(\mathsf{m})}\right|\cdot n_m^{\frac{1}{2}}\left|\frac{\Delta_j^{(\mathsf{m})}}{\widehat{\sigma}_j^{(\mathsf{m})}}\right| + 2\sum_{m=1}^M\left\{\left(\widehat{U}_j^{(\mathsf{m})} - U_j^{(\mathsf{m})}\right)^2 + n_m\left(\frac{\Delta_j^{(\mathsf{m})}}{\widehat{\sigma}_j^{(\mathsf{m})}}\right)^2\right\} \\
=& O_{\mathsf{P}}\left\{n\sum_{m=1}^M (V_j^{(\mathsf{m})})^2\left|(\widehat{\sigma}_j^{(\mathsf{m})})^2 - (\sigma_{0,j}^{(\mathsf{m})})^2\right|\right\} + O_{\mathsf{P}}\left\{\left(n\max_m|V_j^{(\mathsf{m})}| + |\Delta_j^{(\mathsf{m})}|\right)\sum_{m=1}^M|\Delta_j^{(\mathsf{m})}|\right\} \\
\leq & o_{\mathsf{P}}\left\{n\cdot\frac{\log p}{n}\cdot(\log p)^{-1}\right\} + o_{\mathsf{P}}\left\{(n\log p)^{\frac{1}{2}}\cdot\frac{1}{(n\log p)^{\frac{1}{2}}}\right\},
\end{aligned}$$

which indicates that $\breve{\zeta}_j = S_j + o_{\mathsf{P}}(1)$ under the null $\boldsymbol{\beta}_{0,j} = \mathbf{0}$, uniformly for all $j \in \mathcal{H}$. Lemma 2 and the above derivations also indicate that $W_j^{(\mathsf{m})} = U_j^{(\mathsf{m})} + o_{\mathrm{p}}\{(\log p)^{-1/2}\}$.

We next show that

$$\sup_t|\mathsf{P}(S_j \leq t) - \mathsf{P}(\chi_M^2 \leq t)| \to 0, \text{ as } n, p \to \infty.$$

It is equivalent to show that, for any t,

$$\mathsf{P}\Big\{\sum_{m=1}^{M}(U_j^{(\mathsf{m})})^2 \le t\Big\} \to \mathsf{P}(\chi_M^2 \le t). \tag{S10}$$

By Assumptions 1 (i), 3 and 4(a) or 4(b), there exists some constant $c > 0$ such that $\mathsf{P}(\max_{j\in\mathcal{H}}\max_{1\le i\le n_m}|Z_{ij}^{(\mathsf{m})}| \ge \tau_n) = O\{(p+n)^{-2}\}$ with $\tau_n = c\log(p+n)$. Define $U_{j,\tau_n}^{(\mathsf{m})} = n_m^{-\frac{1}{2}}\sum_{i=1}^{n_m} Z_{ij,\tau_n}^{(\mathsf{m})}$, $Z_{ij,\tau_n}^{(\mathsf{m})} = Z_{ij}^{(\mathsf{m})}I(|Z_{ij}^{(\mathsf{m})}| \le \tau_n) - \mathsf{E}\{Z_{ij}^{(\mathsf{m})}I(|Z_{ij}^{(\mathsf{m})}| \le \tau_n)\}$. By Assumptions 3, 4(a) or 4(b), it can be easily seen that

$$\begin{aligned}
\max_{j\in\mathcal{H}} n_m^{-1/2}\sum_{i=1}^{n_m}&\mathsf{E}[|Z_{ij}^{(\mathsf{m})}|I\{|Z_{ij}^{(\mathsf{m})}| \ge \tau_n\}] \\
&\le C n_m^{1/2}\max_{1\le k\le n}\max_{1\le i\le p}\mathsf{E}[|Z_{ij}^{(\mathsf{m})}|I\{|Z_{ij}^{(\mathsf{m})}| \ge \tau_n\}] \\
&\le C n_m^{1/2}(p+n)^{-2},
\end{aligned}$$

for any sufficiently large constant $C > 0$. Hence,

$$\mathsf{P}\Big\{\max_{j\in\mathcal{H}}|U_j^{(\mathsf{m})} - U_{j,\tau_n}^{(\mathsf{m})}| \ge (\log p)^{-2}\Big\} \le \mathsf{P}\Big(\max_{j\in\mathcal{H}}\max_{1\le i\le n_m}|Z_{ij}^{(\mathsf{m})}| \ge \tau_n\Big) = O(p^{-2}). \tag{S11}$$

By the fact that

$$\begin{aligned}
\Big|\max_{j\in\mathcal{H}}\sum_{m=1}^{M}(U_j^{(\mathsf{m})})^2 - \max_{j\in\mathcal{H}}\sum_{m=1}^{M}(U_{j,\tau_n}^{(\mathsf{m})})^2\Big| &\le 2M\max_{j\in\mathcal{H}}\max_{1\le m\le M}|U_{j,\tau_n}^{(\mathsf{m})}|\max_{j\in\mathcal{H}}\max_{1\le m\le M}|U_j^{(\mathsf{m})} - U_{j,\tau_n}^{(\mathsf{m})}| \\
&\quad + M\max_{j\in\mathcal{H}}\max_{1\le m\le M}|U_j^{(\mathsf{m})} - U_{j,\tau_n}^{(\mathsf{m})}|^2,
\end{aligned}$$

it suffices to prove that, for any t, simultaneously for all $j \in \mathcal{H}$,

$$\mathsf{P}\Big\{\sum_{m=1}^{M}(U_{j,\tau_n}^{(\mathsf{m})})^2 \le t\Big\} \to \mathsf{P}(\chi_M^2 \le t). \tag{S12}$$

It follows from Theorem 1 in Zaïtsev (1987) that

$$\mathsf{P}\Big(\Big|n_m^{-1/2}\sum_{i=1}^{n_m} Z_{ij,\tau_n}^{(\mathsf{m})}\Big| \geq t\Big) \leq 2\bar{\Phi}\{t - \epsilon_{n,p}(\log p)^{-1})\} + c_1 \exp\Bigg\{-\frac{n_m^{1/2}\epsilon_{n,p}}{c_2\tau_n(\log p)}\Bigg\}, \tag{S13}$$

and that

$$\mathsf{P}\Big(\Big|n_m^{-1/2}\sum_{i=1}^{n_m} Z_{ij,\tau_n}^{(\mathsf{m})}\Big| \geq t\Big) \geq 2\bar{\Phi}\{t + \epsilon_{n,p}(\log p)^{-1})\} - c_1 \exp\Bigg\{-\frac{n_m^{1/2}\epsilon_{n,p}}{c_2\tau_n(\log p)}\Bigg\}, \tag{S14}$$

where $c_1 > 0$ and $c_2 > 0$ are constants, $\epsilon_{n,p} \to 0$ which will be specified later. Because $\log p = o(n^{1/C'})$ and $M \leq C \log p$ for some constants $C > 0$ and $C' > 6$, by Lemma S4, we let $\epsilon_{n,p} = O\{(\log p)^{(6-C'')/2}\}$ for some constant $C'' \in (6, C')$. This yields that

$$c_1 \exp\Bigg\{-\frac{n_m^{1/2}\epsilon_{n,p}}{c_2\tau_n(\log p)}\Bigg\} = O(p^{-B})$$

for sufficiently large $B > 0$, and

$$\mathsf{P}\Big\{\sum_{m=1}^{M}(U_{j,\tau_n}^{(\mathsf{m})})^2 \geq t\Big\} = (1 + o(1))\mathsf{P}(\chi_M^2 \geq t). \tag{S15}$$

Hence (S12) is proved.

□

S1.4 Proof of Theorem 2

Proof. Recall that $\mathcal{N}_j = \bar{\Phi}^{-1}\Big\{\mathbb{F}_M(\breve{\zeta}_j)/2\Big\}$. We shall first show that

$$\mathsf{P}\Big[\sum_{j\in\mathcal{H}_0} I\{\mathcal{N}_j \geq (2\log q)^{1/2}\} = 0\Big] \to 1 \quad \text{as } (n,p) \to \infty,$$

and then we focus on the event that $\hat{t}$ in (5) exists. Then we will show the FDP result by dividing the null set into small subsets and controlling the variance of $R_0(t)$ for each subset.

The FDR result will follow as well. To this end, we first note that

$$\mathsf{P}\Big[\sum_{j\in\mathcal{H}_0} I\{\mathcal{N}_j \geq (2\log q)^{1/2}\} \geq 1\Big] \leq q_0 \max_{j\in\mathcal{H}_0} \mathsf{P}\{\mathcal{N}_j \geq (2\log q)^{1/2}\},$$

and that, $\mathsf{P}\{\max_{j\in\mathcal{H}_0} |\breve{\zeta}_j - S_j| = o(1)\} = 1$. Then based on Lemma S4, equations (S13), (S14), (S10) and (S15) in the proof of Theorem 1, we have

$$\mathsf{P}\Big[\sum_{j\in\mathcal{H}_0} I\{\mathcal{N}_j \geq (2\log q)^{1/2}\} \geq 1\Big] \leq q_0 G\{(2\log q)^{1/2}\}\{1+o(1)\} + o(1) = o(1),$$

where $G(t) = 2\bar{\Phi}(t)$. Hence, we focus on the event $\{\hat{t}$ exists in the range $[0, (2\log q - 2\log\log q)^{1/2}]\}$. By definition of $\hat{t}$, it is easy to show that

$$\frac{2\{1-\Phi(\hat{t})\}q}{\max\{\sum_{i\in\mathcal{H}} I(\mathcal{N}_j \geq \hat{t}), 1\}} = \alpha.$$

Let $t_q = (2\log q - 2\log\log q)^{1/2}$. It suffices to show that

$$\sup_{0\leq t\leq t_q} \left| \frac{\sum_{j\in\mathcal{H}_0}\{I(\mathcal{N}_j \geq t) - G(t)\}}{qG(t)} \right| \to 0,$$

in probability. Let $0 \leq t_0 < t_1 < \cdots < t_b = t_q$ such that $t_\iota - t_{\iota-1} = v_q$ for $1 \leq \iota \leq b-1$ and $t_b - t_{b-1} \leq v_q$, where $v_q = \{\log q(\log_4 q)\}^{-1/2}$. Thus we have $b \sim t_q/v_q$. For any t such that $t_{\iota-1} \leq t \leq t_\iota$, we have

$$\frac{\sum_{j\in\mathcal{H}_0} I(\mathcal{N}_j \geq t_\iota)}{q_0 G(t_\iota)} \frac{G(t_\iota)}{G(t_{\iota-1})} \leq \frac{\sum_{j\in\mathcal{H}_0} I(\mathcal{N}_j \geq t)}{q_0 G(t)} \leq \frac{\sum_{j\in\mathcal{H}_0} I(\mathcal{N}_j \geq t_{\iota-1})}{q_0 G(t_{\iota-1})} \frac{G(t_{\iota-1})}{G(t_\iota)}.$$

Hence, it is enough to show that

$$\max_{0\leq\iota\leq b} \left| \frac{\sum_{j\in\mathcal{H}_0}\{I(\mathcal{N}_j \geq t_\iota) - G(t_\iota)\}}{qG(t_\iota)} \right| \to 0,$$

in probability. Define $F_j = \sum_{1\leq m\leq M} (U^{(\mathrm{m})}_{j,\tau_n})^2$ and $M_j = \bar{\Phi}^{-1}\left\{\mathbb{F}_M(F_j)/2\right\}$. By equation (S11),

we have $\max_{j\in\mathcal{H}_0}|S_j - F_j| = o_{\mathrm{p}}(1)$. Note that, by Lemma S4, we have

$$\mathsf{P}\{\chi^2_M \geq t + o(1)\}/\mathsf{P}(\chi^2_M \geq t) = 1 + o(1),$$

for any t, and that $G[t + o\{(\log q)^{-1/2}\}]/G(t) = 1 + o(1)$ uniformly in $0 \leq t \leq (2\log q)^{1/2}$. Thus, by equations (S13) and (S14), it suffices to prove that

$$\max_{0\leq\iota\leq b}\left|\frac{\sum_{j\in\mathcal{H}_0}\{I(M_j \geq t_\iota) - G(t_\iota)\}}{q_0 G(t_\iota)}\right| \to 0$$

in probability. Note that

$$\mathsf{P}\left[\max_{0\leq\iota\leq b}\left|\frac{\sum_{j\in\mathcal{H}_0}\{I(M_j \geq t_\iota) - G(t_\iota)\}}{q_0 G(t_\iota)}\right| \geq \epsilon\right] \leq \sum_{\iota=1}^{b}\mathsf{P}\left[\left|\frac{\sum_{j\in\mathcal{H}_0}\{I(M_j \geq t_\iota) - G(t_\iota)\}}{q_0 G(t_\iota)}\right| \geq \epsilon\right]$$
$$\leq \frac{1}{v_q}\int_0^{t_q}\mathsf{P}\left\{\left|\frac{\sum_{j\in\mathcal{H}_0} I(M_j \geq t)}{q_0 G(t)} - 1\right| \geq \epsilon\right\}dt + \sum_{\iota=b-1}^{b}\mathsf{P}\left[\left|\frac{\sum_{j\in\mathcal{H}_0}\{I(M_j \geq t_\iota) - G(t_\iota)\}}{q_0 G(t_\iota)}\right| \geq \epsilon\right].$$

Thus, it suffices to show, for any $\epsilon > 0$,

$$\int_0^{t_q}\mathsf{P}\left[\left|\frac{\sum_{j\in\mathcal{H}_0}\{I(M_j \geq t) - \mathsf{P}(M_j \geq t)\}}{q_0 G(t)}\right| \geq \epsilon\right]dt = o(v_q). \tag{S16}$$

Note that

$$\mathsf{E}\left|\frac{\sum_{j\in\mathcal{H}_0}\{I(M_j \geq t) - \mathsf{P}(M_j \geq t)\}}{q_0 G(t)}\right|^2 = \frac{\sum_{j_1,j_2\in\mathcal{H}_0}\{\mathsf{P}(M_{j_1} \geq t, M_{j_2} \geq t) - \mathsf{P}(M_{j_1} \geq t)\mathsf{P}(M_{j_2} \geq t)\}}{q_0^2 G^2(t)}.$$

Let $[v^{(\mathsf{m})}_{i,j}]_{p\times p} = \mathbb{U}^{(\mathsf{m})}_0\mathbb{J}^{(\mathsf{m})}_0\mathbb{U}^{(\mathsf{m})}_0$ and $\xi^{(\mathsf{m})}_{i,j} = v^{(\mathsf{m})}_{i,j}/(v^{(\mathsf{m})}_{i,i}v^{(\mathsf{m})}_{j,j})^{1/2}$ for $i, j = 1, \ldots, p$. By Assumption 1 and $\mathbb{U}^{(\mathsf{m})}_0 = [\mathbb{H}^{(\mathsf{m})}_0]^{-1}$, we have $C_\Lambda^{-1} \leq \Lambda_{\min}(\mathbb{U}^{(\mathsf{m})}_0\mathbb{J}^{(\mathsf{m})}_0\mathbb{U}^{(\mathsf{m})}_0) \leq \Lambda_{\max}(\mathbb{U}^{(\mathsf{m})}_0\mathbb{J}^{(\mathsf{m})}_0\mathbb{U}^{(\mathsf{m})}_0) \leq C_\Lambda$. For some small enough constant $\gamma > 0$, define

$$\Gamma_j(\gamma) = \{i : |v^{(\mathsf{m})}_{ij}| \geq (\log q)^{-2-\gamma},\ \text{for some } m = 1, \ldots, M\}.$$

It yields that $\max_{j\in\mathcal{H}_0}|\Gamma_j(\gamma)| = o(q^\tau)$ for any $\tau > 0$, and that $\max_{i<j}|\xi^{(\mathsf{m})}_{i,j}| \le \xi$ for some constant $\xi \in (0,1)$.

We divide the indices $j_1, j_2 \in \mathcal{H}_0$ into three subsets: $\mathcal{H}_{01} = \{j_1, j_2 \in \mathcal{H}_0, j_1 = j_2\}$, $\mathcal{H}_{02} = \{j_1, j_2 \in \mathcal{H}_0, j_1 \neq j_2, j_1 \in \Gamma_{j_2}(\gamma), \text{ or } j_2 \in \Gamma_{j_1}(\gamma)\}$, which contains the highly correlated pairs, and $\mathcal{H}_{03} = \mathcal{H}_0 \setminus (\mathcal{H}_{01} \cup \mathcal{H}_{02})$. Then we have

$$\frac{\sum_{j_1,j_2\in\mathcal{H}_{01}}\{\mathsf{P}(M_{j_1} \ge t, M_{j_2} \ge t) - \mathsf{P}(M_{j_1} \ge t)\mathsf{P}(M_{j_2} \ge t)\}}{q_0^2 G^2(t)} \le \frac{C}{q_0 G(t)}. \tag{S17}$$

For the subset $\mathcal{H}_{03}$, in which M_{j_1} and M_{j_2} are weakly correlated with each other. Recall that $F_j = \sum_{1\le m\le M}(U^{(\mathsf{m})}_{j,\tau_n})^2$ and $M_j = \bar{\Phi}^{-1}\left\{\mathbb{F}_M(F_j)/2\right\}$. Then for all $j_1, j_2 \in \mathcal{H}_{03}$,

$$\mathsf{P}(M_{j_1} \ge t, M_{j_2} \ge t) = \mathsf{P}\left\{\sum_{1\le m\le M}(U^{(\mathsf{m})}_{j_1,\tau_n})^2 \ge \mathbb{F}_M^{-1}(G(t)), \sum_{1\le m\le M}(U^{(\mathsf{m})}_{j_2,\tau_n})^2 \ge \mathbb{F}_M^{-1}(G(t))\right\}$$

Similarly as (S13) and (S14), by choosing $\epsilon_{n,p} = 1/(\log p)^2$, based on the condition that $\log p = o(n^{1/10})$, it is easy to check that,

$$c_1 \exp\left\{-\frac{n_m^{1/2}\epsilon_{n,p}}{c_2\tau_n(\log p)}\right\} = O(p^{-B})$$

for sufficiently large $B > 0$, and we have

$$\begin{aligned}
&\mathsf{P}(M_{j_1} \ge t, M_{j_2} \ge t)\\
&\quad\le \mathsf{P}\left\{\sum_{1\le m\le M}(|Z^{(m)}_{j_1}| + \tfrac{\epsilon_{n,p}}{\log p})^2 \ge \mathbb{F}_M^{-1}(G(t)), \sum_{1\le m\le M}(|Z^{(m)}_{j_2}| + \tfrac{\epsilon_{n,p}}{\log p})^2 \ge \mathbb{F}_M^{-1}(G(t))\right\}\\
&\qquad+O(p^{-B+1}),
\end{aligned}$$

where $\{Z^{(m)}_{j_1}, m = 1,\ldots,M\}$ and $\{Z^{(m)}_{j_2}, m = 1,\ldots,M\}$ are standard normal random variables, and their correlations are of the order $O\{(\log q)^{-2-\gamma}\}$. By Lemma S4, it is easy to

obtain that $\max_{1\le j\le p} F_j = o\{(\log p)^{1+\epsilon}\}$ for any sufficiently small constant $\epsilon > 0$. Hence,

$$\begin{aligned}&\mathsf{P}(M_{j_1} \ge t, M_{j_2} \ge t)\\ &\quad\le \mathsf{P}\left\{\sum_{1\le m\le M}(Z_{j_1}^{(m)})^2 \ge \mathbb{F}_M^{-1}(G(t)) - \epsilon_{n,p}(\log p)^{\epsilon}, \sum_{1\le m\le M}(Z_{j_2}^{(m)})^2 \ge \mathbb{F}_M^{-1}(G(t)) - \epsilon_{n,p}(\log p)^{\epsilon}\right\}\\ &\qquad+O(p^{-B+1}).\end{aligned}$$

On the other hand, we also have

$$\begin{aligned}&\mathsf{P}(M_{j_1} \ge t, M_{j_2} \ge t)\\ &\quad\ge \mathsf{P}\left\{\sum_{1\le m\le M}(Z_{j_1}^{(m)})^2 \ge \mathbb{F}_M^{-1}(G(t)) + \epsilon_{n,p}(\log p)^{\epsilon}, \sum_{1\le m\le M}(Z_{j_2}^{(m)})^2 \ge \mathbb{F}_M^{-1}(G(t)) + \epsilon_{n,p}(\log p)^{\epsilon}\right\}\\ &\qquad-O(p^{-B+1}).\end{aligned}$$

Since $\sum_{1\le m\le M}(Z_{j_1}^{(m)})^2$ and $\sum_{1\le m\le M}(Z_{j_2}^{(m)})^2$ are chi-squared random variables, we can transform them back to standard normal variables. By Lemma S4, we have

$$\mathbb{F}_M(\mathbb{F}_M^{-1}(G(t)) + \epsilon_{n,p}(\log p)^{\epsilon}) = (1 + \epsilon_{n,p}(\log p)^{\epsilon})G(t).$$

Then by the fact that $\epsilon_{n,p} = (\log p)^{-2}$,

$$G(t(1 + O(\epsilon_{n,p}/(\log p)^{1-\epsilon}))) = (1 + O((\log p)^{\epsilon}\epsilon_{n,p}))G(t),$$

uniformly in $0 \le t \le (2\log q)^{-1/2}$, and by the correlation assumptions, we have

$$\max_{j_1,j_2\in\mathcal{H}_{03}} \mathsf{P}(M_{j_1} \ge t, M_{j_2} \ge t) = [1 + O\{(\log q)^{-1-\gamma}\}]G^2(t).$$

Thus we have

$$\frac{\sum_{j_1,j_2\in\mathcal{H}_{03}}\{\mathsf{P}(M_{j_1} \ge t, M_{j_2} \ge t) - \mathsf{P}(M_{j_1} \ge t)\mathsf{P}(M_{j_2} \ge t)\}}{q_0^2 G^2(t)} = O\{(\log q)^{-1-\gamma}\}. \tag{S18}$$

Similarly as the above calculations for $\mathcal{H}_{03}$, by Lemma S6 and the condition that $\log p = o(n^{1/10})$, we have

$$\frac{\sum_{j_1,j_2\in\mathcal{H}_{02}}\{\mathsf{P}(M_{j_1}\geq t, M_{j_2}\geq t)-\mathsf{P}(M_{j_1}\geq t)\mathsf{P}(M_{j_2}\geq t)\}}{q_0^2G^2(t)} \leq C\frac{q^{1+\tau}t^{-2}\exp\{-t^2/(1+\xi_1)\}}{q^2G^2(t)} \leq \frac{C}{q^{1-\tau}\{G(t)\}^{2\xi_1/(1+\xi_1)}}, \tag{S19}$$

where ξ_1 is a constant that satisfies $0<\xi<\xi_1<1$.

Therefore, by combining (S17), (S18) and (S19), Equation (S16) follows.

□

S1.5 Proof of Theorem 3

Proof. Note that, by the assumptions of Theorem 3, we have, with probability tending to 1,

$$\sum_{j\in\mathcal{H}} I\{\mathcal{N}_j\geq(2\log q)^{1/2}\}\geq\{1/(\pi^{1/2}\alpha)+\delta\}(\log q)^{1/2}.$$

Therefore, with probability going to one, we have

$$\frac{q}{\sum_{j\in\mathcal{H}} I\{\mathcal{N}_j\geq(2\log q)^{1/2}\}}\leq q\{1/(\pi^{1/2}\alpha)+\delta\}^{-1}(\log q)^{-1/2}.$$

Recall that $t_q=(2\log q-2\log\log q)^{1/2}$. By the fact that $\bar{\Phi}(t_q)\sim 1/\{(2\pi)^{1/2}t_q\}\exp(-t_q^2/2)$, we have $\mathsf{P}(1\leq\hat{t}\leq t_q)\to 1$ according to the definition of $\hat{t}$ in (5). That is, we have

$$\mathsf{P}(\hat{t}\ \textsf{exists in}\ [0,t_q])\to 1.$$

Hence, Theorem 3 is proved based on the proof of Theorem 2.

□

S2. TECHNICAL LEMMAS

In this section, we collect technical lemmas that were used in the previous proofs.

Lemma S1. *Under assumptions of Lemma 1, there exists constants* $c_3, c_3', C_3 > 0$ *and* $\lambda_n^{(\mathsf{m})} \asymp (\log p/n)^{\frac{1}{2}}$ *that when* $n_m \geq c_3 s \log p$, *with probability at least* $1 - c_3' M/p$, *the local LASSO estimator satisfies*

$$\|\widehat{\boldsymbol{\beta}}^{(\mathsf{m})}_{[\text{-}k,\text{-}k']} - \boldsymbol{\beta}_0^{(\mathsf{m})}\|_1 \leq C_3 s \sqrt{\frac{\log p}{n_m}} \quad \text{and} \quad \|\widehat{\boldsymbol{\beta}}^{(\mathsf{m})}_{[\text{-}k,\text{-}k']} - \boldsymbol{\beta}_0^{(\mathsf{m})}\|_2^2 \leq \frac{C_3 s \log p}{n_m}$$

for all $m \in [M]$.

Proof. By Assumption 2, the conditional variance of $\mathbf{Y}_i^{(\mathsf{m})}$ given $\mathbf{X}_i^{(\mathsf{m})}$ is upper-bounded by C_u. Then under Assumptions 1-3 and 4(a) or 4(b), Lemma S1 is a result of Section 4.4 in Negahban et al. (2012).

□

Lemma S2. *Under Assumptions in Lemma 1, for any constant* $t > 0$ *and given* $\boldsymbol{\beta}^{(\bullet)}$ *satisfying* $\|\boldsymbol{\beta}^{(\mathsf{m})} - \boldsymbol{\beta}_0^{(\mathsf{m})}\|_2 = o(1)$ *for* $m \in [M]$, *there exists constants* $C_2, c_2 > 0$ *and* $\phi_0 > 0$ *such that: as* $N \geq C_2 M s \log p$, *with probability at least* $1 - c_2 M/p$, $\mathscr{C}_{\mathsf{RE}}$ *is satisfied for* $\widehat{\mathbb{H}}^{(\bullet)}_{\boldsymbol{\beta}^{(\bullet)}} = \mathsf{diag}\{\widehat{\mathbb{H}}^{(1)}_{\boldsymbol{\beta}^{(1)}}, \ldots, \widehat{\mathbb{H}}^{(\mathsf{M})}_{\boldsymbol{\beta}^{(\mathsf{M})}}\}$ *on any* $|\mathcal{S}| \leq s$ *with parameter* $\phi_0\{t, \mathcal{S}, \widehat{\mathbb{H}}^{(\bullet)}_{\boldsymbol{\beta}^{(\bullet)}}\} \geq \phi_0$.

Proof. By Assumption 4(a) or 4(b), $\mathbf{X}_i^{(\mathsf{m})}$ is sub-gaussian with covariance matrix of eigenvalues bounded away from 0 and ∞. By Lemma S5, $\mathbb{H}^{(\mathsf{m})}_{\boldsymbol{\beta}^{(\mathsf{m})}}$ has bounded eigenvalues away from 0 and ∞. Then we can refer to Negahban et al. (2012) (restricted strong convexity) for the proof of Lemma S2. □

Lemma S3. *Under the assumptions of Lemma 2, there exists*

$$\tau \asymp \frac{M^{\frac{1}{2}}(M + \log p)^{\frac{1}{2}}}{N^{\frac{1}{2}}}$$

such that, with probability converging to 1, *the group dantzig selector type problem (4) has a feasible solution with* $\max_m \|\widehat{\boldsymbol{u}}^{(\mathsf{m})}_{j,[k]}\|_1$ *bounded by some absolute constant for all* $j \in \{2, ..., p\}$, $m \in [M]$ *and* $k \in [K]$.

Proof. For simplicity, we use $\widetilde{\boldsymbol{u}}_j^{(\mathsf{m})}$ to represent the jth row of the inverse of the population covariance matrix $\mathbb{U}^{(\mathsf{m})}_{\widetilde{\boldsymbol{\beta}}^{(\bullet)}_{[\text{-}k]}} = [\mathbb{H}^{(\mathsf{m})}_{\widetilde{\boldsymbol{\beta}}^{(\bullet)}_{[\text{-}k]}}]^{-1}$, weighted with the plugged-in estimator $\widetilde{\boldsymbol{\beta}}^{(\bullet)}_{[\text{-}k]}$ and let $\widetilde{\boldsymbol{u}}_j^{(\bullet)} = (\widetilde{\boldsymbol{u}}_j^{(1)\mathsf{T}}, \dots, \widetilde{\boldsymbol{u}}_j^{(\mathsf{M})\mathsf{T}})^{\mathsf{T}}$. First, we prove that there exists $\tau \asymp \sqrt{M(\log p + M)/N}$, with probability converging to 1, $\widetilde{\boldsymbol{u}}_j^{(\mathsf{m})}$ belongs to the feasible set of (4) for all $j = 2, \dots, p$. Since $\|\widetilde{\boldsymbol{\beta}}^{(\bullet)}_{[\text{-}k]} - \boldsymbol{\beta}_0^{(\mathsf{m})}\|_2 = o_{\mathsf{P}}(1)$ by Lemma 1 and $s = o\{N[M(\log p + M)]^{-1}\}$, by Lemma S5, we have $\mathbf{X}^{(\mathsf{m})}_{i,\widetilde{\boldsymbol{\beta}}^{(\bullet)}_{[\text{-}k]}}$ is sub-gaussian given $\widetilde{\boldsymbol{\beta}}^{(\bullet)}_{[\text{-}k]}$, with probability converging to 1. Then there exists constant $C_9 > 0$ that with probability converging to 1,

$$\|\widetilde{\mathbb{H}}^{(\bullet)}_{[k]}\widetilde{\boldsymbol{u}}_j^{(\bullet)} - \boldsymbol{e}_j^{(\bullet)}\|_{2,\infty} \leq C_9\sqrt{\frac{M(\log p + M)}{N}},$$

which indicates that problem (4) has feasible solution. Since (4) minimizes $\max_{m\in[M]} \|\boldsymbol{u}_j^{(\mathsf{m})}\|_1$ and $\widetilde{\boldsymbol{u}}_j^{(\bullet)}$ belongs to the feasible set, we have $\max_{m\in[M]} \|\widehat{\boldsymbol{u}}^{(\mathsf{m})}_{j,[k]}\|_1 \leq \max_{m\in[M]} \|\widetilde{\boldsymbol{u}}_j^{(\mathsf{m})}\|_1$ and then the boundness of $\max_{m\in[M]} \|\widehat{\boldsymbol{u}}^{(\mathsf{m})}_{j,[k]}\|_1$ follows from Assumption 1 (i).

□

Lemma S4 (Zolotarev (1961))**.** *Let $\boldsymbol{Y}$ be a nondegenerate gaussian mean zero random variable (r.v.) with covariance operator $\boldsymbol{\Sigma}$. Let σ^2 be the largest eigenvalue of $\boldsymbol{\Sigma}$ and d be the dimension of the corresponding eigenspace. Let σ_i^2, $1 \leq i < d'$, be the positive eigenvalues of $\boldsymbol{\Sigma}$ arranged in a nonincreasing order and taking into account the multiplicities. Further, if $d' < \infty$, put $\sigma_i^2 = 0$, $i \geq d'$. Let $H(\boldsymbol{\Sigma}) := \prod_{i=d+1}^{\infty}(1 - \sigma_i^2/\sigma^2)^{-1/2}$. Then for $y > 0$,*

$$P\{\|\boldsymbol{Y}\| > y\} \sim 2A\sigma^2 y^{d-2}\exp(-y^2/(2\sigma^2)), \text{ as } y \to \infty,$$

where $A := (2\sigma^2)^{-d/2}\Gamma^{-1}(d/2)H(\boldsymbol{\Sigma})$ with $\Gamma(\cdot)$ the gamma function.

Lemma S5. *Under the same assumptions of Lemma 1, for any $m \in [M]$ and any given $\boldsymbol{\beta}^{(\mathsf{m})}$ satisfying $\|\boldsymbol{\beta}^{(\mathsf{m})} - \boldsymbol{\beta}_0^{(\mathsf{m})}\|_2 = o(1)$, there exists constant $C_0 > 0$ such that*

$$C_0^{-1} \leq \Lambda_{\min}\left\{\mathbb{H}^{(\mathsf{m})}_{\boldsymbol{\beta}^{(\mathsf{m})}}\right\} \leq \Lambda_{\max}\left\{\mathbb{H}^{(\mathsf{m})}_{\boldsymbol{\beta}^{(\mathsf{m})}}\right\} \leq C_0.$$

Proof. For any $\boldsymbol{x} \in \mathbb{R}^p$ satisfying $\|\boldsymbol{x}\|_2 = 1$, by Assumption 2, we have

$$|\boldsymbol{x}^\mathsf{T}\mathbb{H}^{(\mathsf{m})}_{\boldsymbol{\beta}^{(\mathsf{m})}_0}\boldsymbol{x} - \boldsymbol{x}^\mathsf{T}\mathbb{H}^{(\mathsf{m})}_{\boldsymbol{\beta}^{(\mathsf{m})}}\boldsymbol{x}| = |\mathsf{E}(\boldsymbol{x}^\mathsf{T}\mathbf{X}_i^{(\mathsf{m})})^2\{\ddot{\phi}(\mathbf{X}_i^{(\mathsf{m})\mathsf{T}}\boldsymbol{\beta}_0^{(\mathsf{m})}) - \ddot{\phi}(\mathbf{X}_i^{(\mathsf{m})\mathsf{T}}\boldsymbol{\beta}^{(\mathsf{m})})\}|$$

$$\leq \mathsf{E}(\boldsymbol{x}^\mathsf{T}\mathbf{X}_i^{(\mathsf{m})})^2 C_L|\mathbf{X}_i^{(\mathsf{m})}\{\boldsymbol{\beta}_0^{(\mathsf{m})\mathsf{T}} - \boldsymbol{\beta}^{(\mathsf{m})\mathsf{T}}\}| \leq C_L\left(\mathsf{E}[\boldsymbol{x}^\mathsf{T}\mathbf{X}_i^{(\mathsf{m})}]^4 \cdot \mathsf{E}[\mathbf{X}_i^{(\mathsf{m})}\{\boldsymbol{\beta}_0^{(\mathsf{m})\mathsf{T}} - \boldsymbol{\beta}^{(\mathsf{m})\mathsf{T}}\}]^2\right)^{\frac{1}{2}}.$$

By Assumption 1 (i) and Assumption 4(a) or 4(b), we have that $\mathsf{E}[\boldsymbol{x}^\mathsf{T}\mathbf{X}_i^{(\mathsf{m})}]^4$ is bounded by some absolute constant for all $\boldsymbol{x}$ and $\mathsf{E}[\mathbf{X}_i^{(\mathsf{m})}\{\boldsymbol{\beta}_0^{(\mathsf{m})\mathsf{T}} - \boldsymbol{\beta}^{(\mathsf{m})\mathsf{T}}\}]^2 = o(1)$ since $\|\boldsymbol{\beta}^{(\mathsf{m})} - \boldsymbol{\beta}_0^{(\mathsf{m})}\|_2 = o(1)$. Thus, we have

$$|\boldsymbol{x}^\mathsf{T}\mathbb{H}^{(\mathsf{m})}_{\boldsymbol{\beta}^{(\mathsf{m})}_0}\boldsymbol{x} - \boldsymbol{x}^\mathsf{T}\mathbb{H}^{(\mathsf{m})}_{\boldsymbol{\beta}^{(\mathsf{m})}}\boldsymbol{x}| = o(1),$$

and the conclusion follows directly from Assumption 1 (i). □

Lemma S6 (Berman (1962))**.** *If X and Y have a bivariate normal distribution with expectation zero, unit variance and correlation coefficient ρ, then*

$$\lim_{c\to\infty} \frac{P(X > c, Y > c)}{\{2\pi(1-\rho)^{1/2}c^2\}^{-1}\exp\left(-\frac{c^2}{1+\rho}\right)(1+\rho)^{1/2}} = 1,$$

uniformly for all ρ such that $|\rho| \leq \delta$, for any δ, $0 < \delta < 1$.

S3. ADDITIONAL NUMERICAL RESULTS

In this section, we present additional numerical results for binary hidden markov model. Figure S1 illustrates that, the false discovery rate and power results for hidden markov model design has almost the same pattern as those of the Gaussian design.

Figure S1: The empirical FDR and power of our DSILT method, the one–shot approach and the ILMA method under the binary HMM design, with $\alpha = 0.1$. The horizontal axis represents the overall signal magnitude μ.

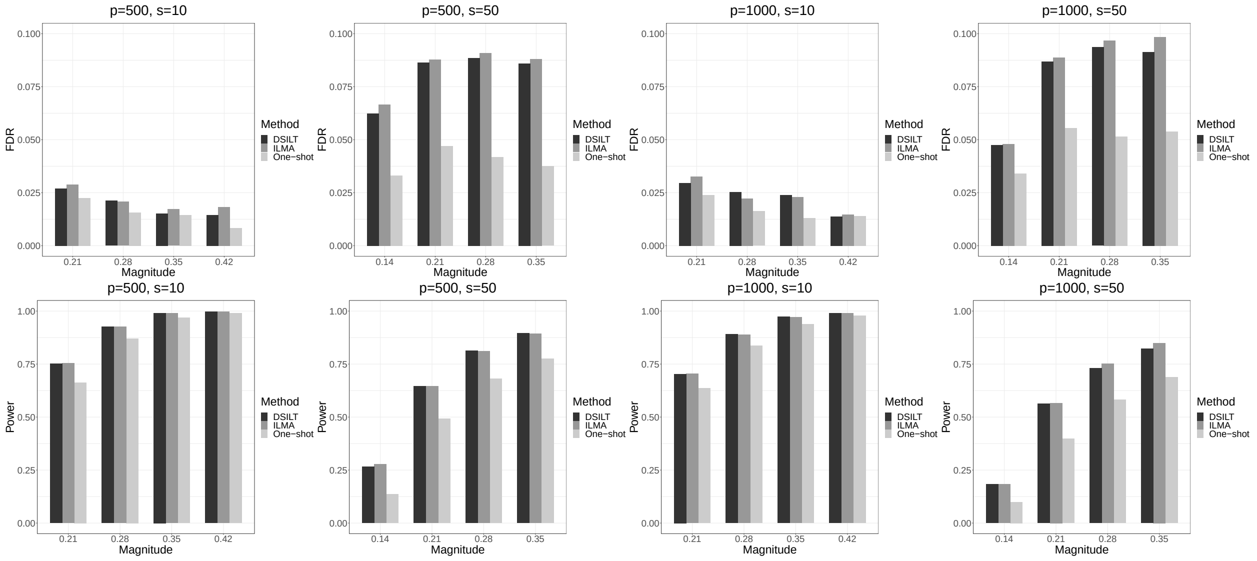

REFERENCES

Berman, S. M. (1962). A law of large numbers for the maximum in a stationary Gaussian sequence. *Ann. Math. Statist. 33*, 93–97.

Cai, T., M. Liu, and Y. Xia (2019). Individual data protected integrative regression analysis of high-dimensional heterogeneous data. *arXiv preprint arXiv:1902.06115*.

Huang, J. and T. Zhang (2010). The benefit of group sparsity. *The Annals of Statistics 38*(4), 1978–2004.

Kuchibhotla, A. K. and A. Chakrabortty (2018). Moving beyond sub-gaussianity in high-dimensional statistics: Applications in covariance estimation and linear regression. *arXiv preprint arXiv:1804.02605*.

Liu, W. (2013). Gaussian graphical model estimation with false discovery rate control. *Ann. Statist. 41*(6), 2948–2978.

Lounici, K., M. Pontil, S. Van De Geer, A. B. Tsybakov, et al. (2011). Oracle inequalities and optimal inference under group sparsity. *The Annals of Statistics 39*(4), 2164–2204.

Negahban, S. N., P. Ravikumar, M. J. Wainwright, B. Yu, et al. (2012). A unified framework for high-dimensional analysis of m-estimators with decomposable regularizers. *Statistical Science 27*(4), 538–557.

Zaïtsev, A. Y. (1987). On the Gaussian approximation of convolutions under multidimensional analogues of SN Bernstein's inequality conditions. *Probab. Theory Rel. 74*(4), 535–566.

Zolotarev, V. M. (1961). Concerning a certain probability problem. *Theory Probab. Appl. 6*(2), 201–204.
